# Novel 8-Substituted Coumarins That Selectively Inhibit Human Carbonic Anhydrase IX and XII

**DOI:** 10.3390/ijms20051208

**Published:** 2019-03-10

**Authors:** Kerem Buran, Silvia Bua, Giulio Poli, F. Esra Önen Bayram, Tiziano Tuccinardi, Claudiu T. Supuran

**Affiliations:** 1 Department of Pharmaceutical Chemistry, Faculty of Pharmacy, Yeditepe University, Kayisdagi Cad., 34755 Istanbul, Turkey; keremburan@gmail.com; 2 NEUROFARBA Department, Pharmaceutical and Nutraceutical Section, University of Florence, 50019 Sesto Fiorentino, Italy; silvia.bua@unifi.it (S.B.); claudiu.supuran@unifi.it (C.T.S.); 3 Department of Pharmacy, University of Pisa, 56126 Pisa, Italy; giulio.poli@unipi.it

**Keywords:** carbonic anhydrase inhibitor, metalloenzymes, coumarins, docking, molecular modeling

## Abstract

A novel series of 8-substituted coumarin-based compounds, characterized by the presence of alkylpiperazine and arylpiperazine chains, were synthesized and tested for their inhibitory activity against four human carbonic anhydrase (*h*CA) isoforms. All compounds displayed nanomolar potency against the cancer-related *h*CA IX and *h*CA XII; moreover, they were shown to be devoid of any inhibitory activity toward the cytosolic *h*CA I and *h*CA II up to 10 µM concentration in the assay system. Therefore, the synthesized coumarin ligands demonstrated to be potent and selective *h*CA IX/XII inhibitors, and were shown to be as potent as the reference inhibitor acetazolamide against *h*CA XII, with single-digit nanomolar *K*_i_ values. Molecular modeling studies provided a rationale for explaining the selectivity profile of these non-classic *h*CA inhibitors and their interactions with the enzymes, according to their specific mechanism of action, thus paving the way for future structure-based lead optimization studies.

## 1. Introduction

Carbonic anhydrase (CA, EC 4.2.1.1) is a family of metalloenzymes that catalyze the reversible interconversion of carbon dioxide and water to bicarbonate and a proton [1]. In humans, sixteen CA isoforms are present and they all differ for kinetic properties, sub-cellular localization and tissue distribution. The different *h*CAs are involved in many physiological processes such as pH regulation, CO_2_ homeostasis, respiration, bone resorption and tumorigenesis [2]. *h*CA inhibitors (CAIs) are in pharmacological/clinical use for many decades as diuretics and antiglaucoma agents [3,4]. More recently, they have also been evaluated for the imaging, for the treatment of hypoxic tumors [5,6,7] and as anti-obesity agents [8]. CAIs are generally compounds endowed with a suitable zinc-binding group (ZBG) able to chelate the prosthetic zinc ion placed within the binding site of *h*CAs, which is critical for the catalytic activity of these enzymes. The most common ZBGs shared by classic CAIs are represented by sulfonamide moieties or similar structural motifs (such as sulfamides and sulfamates). These groups are particularly effective for endowing small-molecule ligands with high affinity for *h*CAs, since they not only allow a proper coordination of the catalytic zinc ion but also the formation of H-bond interactions with key protein residues placed in the surroundings of the zinc-binding cavity. In fact, these compounds represent the most important classes of CAIs and numerous ligands showing high inhibitory potency have been reported to date [9]. However, due to the high amino acid conservation observed in the different *h*CA isoforms at the level of their catalytic site and adjacent regions, these ligands often present the drawback of being insufficiently selective toward specific therapeutically interesting *h*CAs. In particular, *h*CA IX and *h*CA XII have drawn special attention in the medicinal chemistry field, since these isoforms have been designated as biomarkers and pharmacological targets for various cancer types. As a consequence, a wide area of research is currently focused on the inhibition of these two enzymes [10]. Nevertheless, the development *h*CA IX/XII inhibitors with selectivity over *h*CA I/II, which are ubiquitously distributed and involved in key physiological processes, is still a challenging task, although some example of selective ligands have been reported [11,12]. In this context, non-classical CAIs constitute a particularly valuable resource to seek for isoform specificity and prevent potential off-targets activities, side effects and/or adverse reactions (such as sulphur allergies) connected to the use of sulfurated ligands [13]. Among non-classical CAIs, coumarin derivatives represent a particularly atypical category of *h*CA ligands due to their peculiar mechanism of inhibition, which does not involve the chelation of the prosthetic zinc ion. In fact, upon binding to the catalytic site of *h*CAs, these ligands are hydrolyzed by the esterase activity of the enzymes to the corresponding 2-hydroxy-cinnamic acid derivatives, which thus represent the actual inhibitors. As demonstrated by crystallographic studies, these derivatives bind at the entrance of *h*CAs catalytic pocket hampering the access to the site and blocking the catalytic activity of the enzymes [14,15]. Coumarin-based inhibitors, particularly 7-hydroxycoumarin derivatives and substituted analogues, were shown to be endowed with a good selectivity profile, demonstrating nanomolar inhibition of *h*CA IX and XII, and a much lower potency against *h*CA I and II, with some representative compounds demonstrating also cytotoxic activity in cancer cells [16,17,18,19,20]. With the purpose of expanding the chemical space of available coumarin-based ligands and the corresponding structure activity relationships, in the search for novel potent and selective CAIs, a novel series of 7-hydroxycoumarin derivatives has been synthesized and evaluated for their *h*CA inhibitory activity. All compounds were found to selectively inhibit *h*CA IX and especially *h*CA XII, showing single-digit nanomolar activity for this latter isoform, thus confirming the importance of the coumarin scaffold for the development of selective CAIs.

## 2. Results and Discussion

### 2.1. Synthesis of 7-Hydroxycoumarin Derivatives

**Scheme 1:**
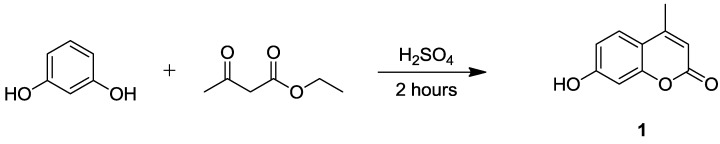
Synthesis of 4-methylumbelliferone (compound **1**).

**Scheme 2:**
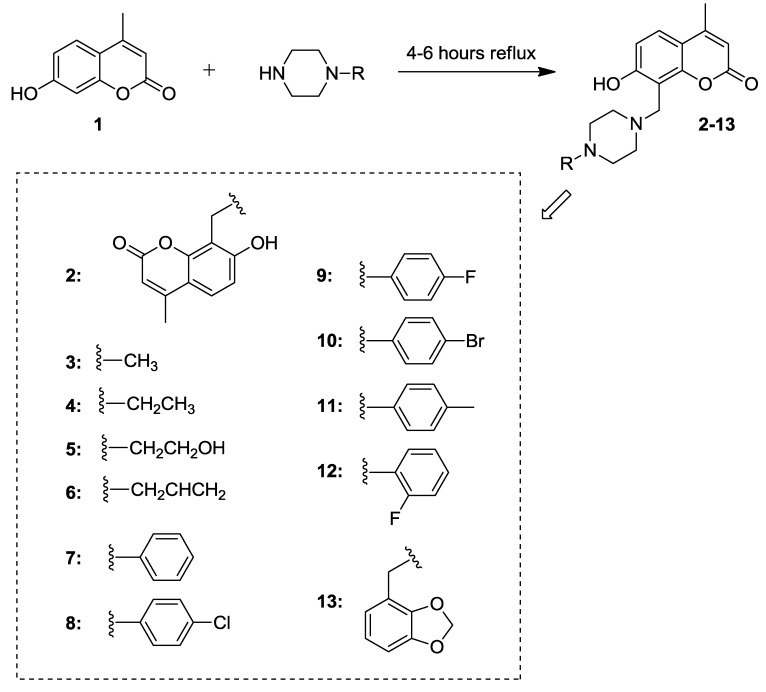
Synthesis of piperazine derivatives **2**–**13**.

Our synthetic efforts aimed at the identification of new coumarin-based CAIs focused on the substitution of position 8 of the central chromen-2-one scaffold. In particular, 4-methylumbelliferone (7-hydroxy-4-methyl-chromen-2-one) (**1**) was initially synthesized starting from resorcinol and ethyl acetoacetate according to the Pechmann reaction (Scheme 1). Subsequently, twelve derivatives of compound **1** functionalized at the position 8 of the bicyclic core were obtained through reaction with a series of five aliphatic and seven aromatic piperazine fragments (Scheme 2).

Compound **1** was characterized with ^1^H-NMR, ^13^C-NMR and LC-MS. The ^1^H-NMR spectrum of this compound presented a doublet at 7.57 ppm (*J* = 8.7 Hz) for the aromatic **H_3_**. Its neighboring proton **H_4_** gave a doublet of doublet (*J*_1_ = 8.7 Hz, *J*_2_ = 2.4 Hz) due to the o-coupling with **H_3_** and m-coupling with **H_6_** that also gave a doublet at 6.68 ppm (*J* = 2.4 Hz). The doublet at 6.10 ppm (*J* = 1.2 Hz) was obtained for the **H_1_** proton, which presents a typical a *cis*-allylic coupling with the methyl moiety bond to the cyclic double bond. Finally, the presence of the methyl was confirmed by the quartet obtained at 2.34 ppm (*J* = 1.2 Hz) and the hydroxyl gave a signal for one proton at 10.5 ppm. All these findings confirmed clearly the successful cyclization of resorcinol to generate the coumarin ring. The synthesized compound **1** was derivatized with a series of five aliphatic and seven aromatic piperazines. Targeted molecules were obtained by binding the heterocyclic moieties at the position 8 of compound **1** using formaldehyde. Yields obtained for the synthesized molecules (**2**–**13**) are given in Table S1. The substitution of the coumarin heterocycle was easy to monitor via ^1^H-NMR as a successful substitution gave a new singlet at around 4.0 ppm that integrated for two protons, corresponding to the methylene spacer between the coumarin and piperazine heterocycles. However, interpreting the signals of the piperazine cycle was not obvious as they can give multiplets or more commonly broad singlets due to eight non-equivalent protons that, in addition to short-range correlation, can present long-range correlations as mentioned in the literature [21]. The spectrum of each compound is detailed in the experimental part and can also be found in the supplementary materials.

### 2.2. Inhibitory Activity against hCA Isoforms

The thirteen total compounds were subjected to a stopped-flow CO_2_ hydrase assay, together with the reference inhibitor acetazolamide (**AAZ**), in order to evaluate their inhibitory activity against *h*CA I, II, IX and XII. As reported in Table 1, all compounds were found to inhibit the cancer-related *h*CA IX and *h*CA XII with K_i_ values in the nanomolar range and showed to be devoid of any hCA I and *h*CA II inhibitory activity up to 10 µM concentration in the assay system. The tested compounds showed a higher affinity for *h*CA XII with respect to *h*CA IX (from 5-fold up to 40-fold). In fact, the activity of the coumarin ligands against *h*CA XII was comparable to that observed for the **AAZ**, with single-digit nanomolar K_i_ values determined for all compounds except for the *o*-fluorophenylpiperazine derivative **12** (K_i_ for hCA XII of 26.4 nM). It is interesting to note that the derivatization of 4-methylumbelliferone (**1**) with both aliphatic and aromatic piperazine fragments apparently seemed to have no influence on *h*CA XII inhibitory activity; this suggests that the corresponding side chains of compounds **2**–**13** might not establish key interactions with *h*CA XII catalytic site able to affect their affinity for the protein. As far as *h*CA IX inhibition is concerned, the activity of the ligands spanned a wider range, with the alkylpiperazine derivatives showing better potencies, on average, than the arylpiperazine compounds. In fact, the K_i_ values determined for the different derivatives ranged from around 300 nM (for compounds **5** and **11**) to the lowest value of 27.1 nM observed for compound **6**, which was found to be as potent as **AAZ** and represented the most active compound of the series. However, derivative **6** still showed an activity profile comparable to that of the parent compound **1**, hence confirming the relatively slight impact of the piperazine substituents on the *h*CA IX/XII inhibitory activity of these ligands.

### 2.3. Molecular Modeling Studies

**Figure 1:**
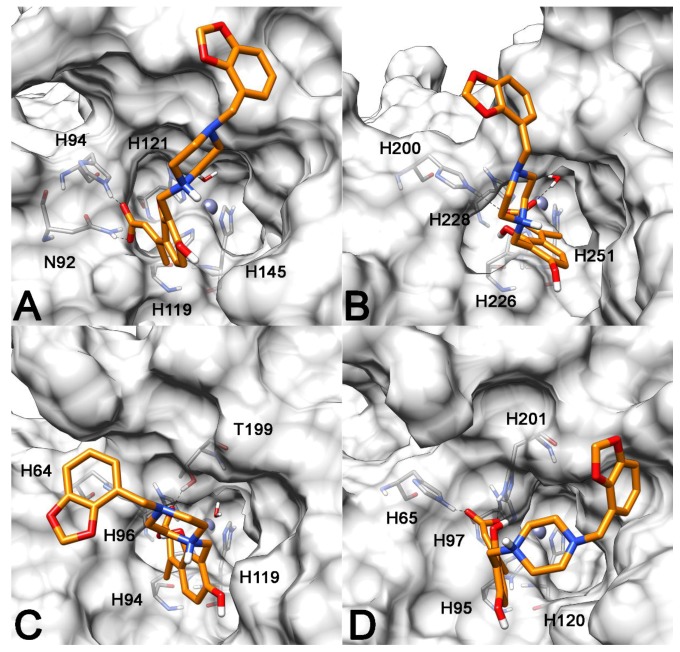
Minimized average structures of compound **13** in hydrolyzed form, in complex with *h*CA XII (**A**); *h*CA IX (**B**); *h*CA II (**C**); and *h*CA I (**D**). Only residues coordinating the zinc ion or forming H-bond with the ligands are shown in cyan. The protein surface is shown in gray. The zinc ion is shown as a gray sphere.

With the aim of figuring out the ligand–protein interaction features at the basis of the selectivity for *h*CA IX and XII over *h*CA I and II demonstrated by the synthesized coumarin derivatives, as well as to get clues about the impact of their piperazine side chains on their activity profile, molecular modeling studies including docking and molecular dynamics (MD) simulations were carried out. Compound **13**, which showed good potency on both *h*CA IX and XII, was taken into account for this study as a reference ligand of the series. Coumarin derivatives are known to undergo hydrolysis upon binding to the catalytic site of *h*CAs, which leads to the formation of 2-hydroxy-cinnamic acids. These compounds keep binding to the enzymes and thus represent the real CAIs, as confirmed by X-ray crystallography [14,15]. For this reason, the 2,4-dihydroxy-cinnamic acid that can be obtained from hydrolysis of compound **13** was docked into the X-ray structures of *h*CA I, II, IX and XII (PDB codes 1AZM, 2AW1, 3IAI and 1JD0, respectively) by employing Autodock4 software (Autodock 4.2, The Scripps Research Institute, La Jolla, CA, USA). Subsequently, the four ligand–protein complexes generated by docking were further analyzed with MD simulation studies (see the Materials and Methods section for details). In the binding modes generated through the whole computational protocol, the ligand was predicted to interact with the four *h*CA isoforms showing a similar disposition of the 2,4-dihydroxy-cinnamic moiety across the protein catalytic site. The carboxylic group of the ligand was oriented toward the hydrophilic half of the enzyme, forming hydrogen bonds with the polar residues located in this part of the binding site (Figure 1), in agreement with the experimentally determined binding mode of 2-hydroxy-cinnamic acid bound to *h*CA II [15]. In particular, the ligand was predicted to interact with all four *h*CA isoforms establishing two different H-bonds, one of which always involved a histidine residue located in the hydrophilic side of the binding cavity, next to the catalytic residues, i.e., H94, H200, H64 and H65 for *h*CA XII, IX, II and I, respectively (see Figure S1 in the Supplementary Materials for the sequence alignment of the four *h*CAs). In *h*CA XII, the second H-bond formed by the ligand was established with N92, while in *h*CA IX the compound was found to interact with the zinc-bound water molecule. Finally, in *h*CA II and *h*CA I, the ligand was predicted to form H-bonds with T199 and its homologue residue H201, respectively. On the contrary, the long piperazine-containing chain connected to the cinnamic moiety showed to be highly solvent exposed and to assume rather different orientations into the binding sites of the four different *h*CAs, without forming significant interactions with the four proteins. In fact, the ligand terminal chain was predicted to interact with the hydrophobic portion of *h*CA I and the polar side of *h*CA II catalytic pockets, while when bound to *h*CA IX and *h*CA XII, the ligand oriented its chain in the middle between the two sides. In all complexes, the piperazine-containing substituent did not show interactions that could substantially contribute to the ligand’s affinity for the *h*CA isoforms. This is consistent with the results of our experimental assays, highlighting the modest impact of the different piperazine fragments on the activity profile of this series of compounds. Although the ligand did not protrude into the inner part of the *h*CAs binding pocket where the zinc ion and the catalytic residues are located, MD simulation results showed that the core scaffold of compound **13** was firmly anchored to the protein thanks to the stable H-bonds formed by the carboxyl group (maintained for almost the whole MD simulations) and could thus easily block the entrance of the catalytic pocket of the four *h*CAs. 

**Figure 2:**
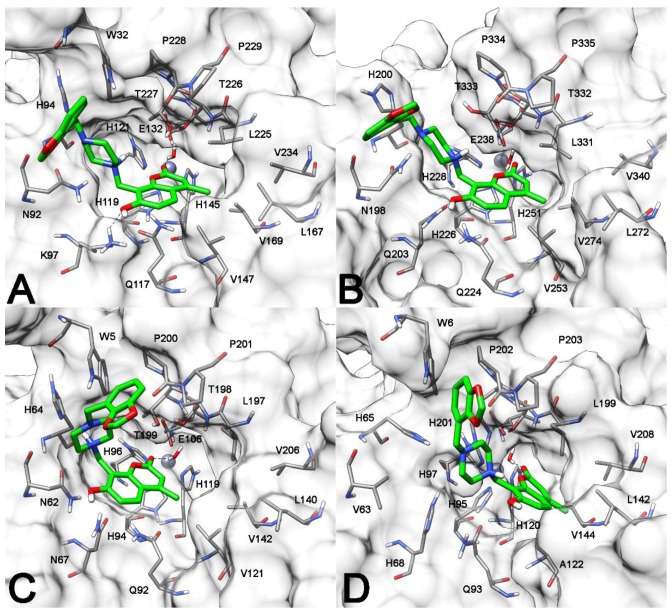
Minimized average structures of compound **13** (green) in complex with *h*CA XII (**A**); *h*CA IX (**B**); *h*CA II (**C**); and *h*CA I (**D**). Ligand–protein H-bonds are shown as black dashed lines, and the protein surface in the proximity of the ligands is shown in grey. The zinc ion is shown as a gray sphere.

Hence, the analysis of the binding modes calculated for the hydrolysis product of compound **13** into the four *h*CA isoforms suggested that the ligand could strongly bind to all the four *h*CAs. Therefore, it was not possible to identify a rationale explaining the selective activity of the ligand and its analogues against *h*CA IX and XII. These results led us to hypothesize that the ligand recognition process, i.e., the binding interactions established by the coumarin derivatives prior to their hydrolysis, could be implied in the selectivity of this class of compounds. It was already hypothesized by Supuran and co-workers that these derivatives, before their hydrolysis, may bind within the CA active site similarly to phenols i.e., by anchoring to the zinc-bound water molecule/hydroxide ion [15]. This specific binding mode has indeed been confirmed for a 2-thioxocoumarine recently co-crystallized with CA II [19]. For this reason, the whole computational protocol based on docking and MD simulation studies was performed considering compound **13** in its native form. Figure 2 shows the binding mode predicted for the ligand into the catalytic site of *h*CA XII. The carbonyl oxygen of the coumarin core forms a hydrogen bond with the water molecule coordinating the prosthetic zinc ion, in agreement with the mechanism of hydrolysis proposed for this series of derivatives [15]. The bicyclic scaffold of the ligand is placed above the side chains of H119, Q117 and K97, forming interactions with these residues. In particular, an NH–π interaction is observed with the amide group of Q117, while the phenolic group of the ligand forms an H-bond with the charged nitrogen of K97. Finally, the 4-methyl group of the coumarin core is placed in a hydrophobic pocket delimited by V147, L167 and L225, thus taking lipophilic contacts with these residues, while the N-substituted piperazine chain primarily forms van der Waals interactions with residues constituting the hydrophilic site of the protein binding site, such as W32, N92 and H94. A very similar binding pose was predicted for the ligand into *h*CA IX: the compound essentially formed the same pattern of interactions observed with *h*CA XII, including the key interaction with the zinc-coordinating structural water, but with the exception of the H-bond with K97 (Figure 2B). In fact, K97 is a non-conserved residue of *h*CA XII, substituted by Q203 in *h*CA IX; nevertheless, the ligand is still able to form an H-bond with the side chain of this latter residue through the phenolic OH group and to form stacking interactions with Q224.

Based on the binding mode predicted for compound **13** into *h*CA XII and IX, the non-conserved residues K97 and Q203 seemed to play a substantial role in ligand–protein recognition, which could also help explain the selectivity for *h*CA XII and IX of this class of compounds. In fact, the analysis of the binding mode predicted for the ligand into *h*CA II revealed that, although the coumarin core of the inhibitor assumes a disposition similar to that showed into the other two CA isoforms, the phenolic OH group is not able to establish any H-bond interaction, due to the presence of the asparagine residue N67 in place of the homolog residues K97 and Q203 present in *h*CA XII and IX, respectively. Precisely because of the shorter side chain of N67 compared to Q203, the residue can only form an NH–π interaction with the aromatic ring of the ligand, which thus loses a key anchoring point to the protein. This also determines a slight shift of the compound core away from the hydrophobic side of the protein binding cavity, thus reducing the lipophilic interactions with V121, L140 and L197. On the contrary, the presence of residue H68 in *h*CA I, in place of the homolog residues K97 and Q203 present in *h*CA XII and IX, respectively, would not only prevent the formation of H-bonds with the ligand but also create a steric hindrance that determines its shift toward the hydrophobic side of the catalytic site. In fact, compound **13** was predicted to bind *h*CA I with the coumarin core closer to the lipophilic residues of the binding site such as V144, L199 and V208. However, this determines the lack of both H-bonds and NH–π interactions with the residues belonging to the hydrophilic side of the protein. The missing H-bonds with the polar residues of *h*CA II and *h*CA I binding sites could be at the basis of the very weak activity demonstrated by compound **13** and its analogues against the two *h*CA isoforms. Moreover, the higher potency against *h*CA XII compared to *h*CA IX observed for this class of compounds could be explained considering the stronger H-bond interaction that the coumarin derivatives can form with the charged residue K97 compared to Q203. Interestingly, these considerations are in agreement with the observation that the structural element that seems to confer *h*CA IX–XII selectivity to this class of compound is the 7-hydroxyl group of the coumarin core, which should be implied in the H-bond with K97 of *h*CA XII and Q203 of *h*CA IX. In fact, compound **1** showed the same selectivity profile of the whole class of derivatives, despite the absence of the long and charged substituent in position 8 of the bicyclic ring.

## 3. Materials and Methods

### 3.1. Chemistry

General procedure for 7-Hydroxy-4-methyl-chromen-2-one (**1**). Resorcinol (1.22 eq) was dissolved in concentrated H_2_SO_4_ (20 mL) at 0 °C, then ethyl acetoacetate (1.0 eq) was slowly added and the mixture was stirred at 0–5 °C for 2 h. At the end of the two hours, the reaction mixture was poured onto ice-water. Then, the solid was washed with water and recrystallized from EtOH. Finally, pure white solid 7-Hydroxy-4-methyl-chromen-2-one was obtained [22].

General procudure for synthesis of 7-hydroxy-4-methyl-8 (piperazines)chromen-2-ones (**2**–**13**). Compound **1** (7-Hydroxy-4-methyl-chromen-2-one; 1.0 eq) was dissolved in 95% EtOH (5 mL), and then piperazine derivative(s) (*R*) (1.0 eq) and formaldehyde (0.2 mL) were added to the reaction medium. The reaction mixture was refluxed for 4–6 h. At the end of the 4–6 h, the reaction medium was cooled and then the solvent was evaporated in vacuo. Pale-yellow oils were obtained and further treated with a little amount of cool acetone. Then, the white solids were crystallized from acetone [23].

*7-Hydroxy-4-methylchromen-2-one* (**1**): FT-IR: (KBr), cm^−1^: 3150–3050 (OH); 3011 (aromatic C–H); 2958 (aliphatic C–H); 1679 (C=O, lactone); 1599 (C=C, aromatic). ^1^H-NMR: (400 MHz, DMSO) δ 10.50 (s, 1H, OH); 7.55 (d, *J* = 8.7, 1H, H_5_); 6.78 (dd, *J*_1_ =8.7 Hz, *J*_2_ = 2.4 Hz, 1H, H_6_); 6.67 (d, *J* = 2.4 Hz, 1H, H_8_); 6.08 (q, *J* = 1.2 Hz, 1H, H_3_); 3.33 (d, *J* = 1.2 Hz 3H, H_9_). ^13^C-NMR: (400 MHz, DMSO) δ 161.5 (C_2_); 160.7, 155.2, 153.9, 127.0, 113.2, 112.4 (C_coumarin-aromatic_); 110.6, 102.6 (C_3-4_); 18.5 (C_9_). LC-MS: rt = 5.71 min, *m/z*, 178.13 [M + H]^+^, Purity: 91%, UV (ACN/Water, λ_max_): 320 nm.

*1,4-bis[(7-Hydroxy-4-methylcoumarin-8-yl)methyl] piperazine* (**2**): FT-IR: (KBr), cm^−1^: 3200–3050 (OH); 3048 (aromatic C–H); 2958 (aliphatic C–H); 1722 (C=O, lactone); 1627 (C=C, alkene); 1602 (C=C, aromatic). ^1^H-NMR (400 MHz, CDCl_3_) δ7.41 (t, *J* = 9.70 Hz, 2H, H_5_); 6.77 (dd, *J*_1_ = 8.70 Hz, *J*_2_ = 15.3 Hz, 2H, H_6_); 6.08 (d, *J* = 6.50 Hz, 2H, H_3_); 4.08 (d, *J* = 15.80 Hz, 4H, H_10_); 2.49 (s, 16H, H_11,12_); 2.38 (s, 6H, H_9_). ^13^C-NMR (400 MHz, CDCl_3_) δ 162.5 (C_2_); 161.2, 153.3, 152.4, 126.8, 113.3, 112.1 (C_coumarin-aromatic_); 110.6, 107.6 (C_3,4_); 53.8, 52.6 (C_11,12_); 51.4 (C_10_); 18.8 (C_10_). LC-MS: rt = 5.10 min, *m/z*, 463.42 [M + H]^+^, Purity: 93%, UV (ACN/Water, λ_max_): 320 nm. 

*7-Hydroxy-4-methyl-8-(4-methylpiperazin-1-ylmethyl)chromen-2-one* (**3**): FT-IR: (KBr), cm^−1^: 3300–3100 (OH); 3071 (aromatic C–H), 1726 (C=O, lactone), 1609 (C=C, nonaromatic), 1580 (Ar C=C). ^1^H-NMR (400 MHz, CDCl_3_) δ 7.40 (d, *J* = 8.7 Hz, 1H, H_5_); 6.75 (d, *J* = 8.7 Hz, 1H, H_6_); 6.07 (q, *J* = 1.1 Hz, 1H, H_3_); 4.06 (s, 2H, H_10_); 2.90–2.40 (m, 8H, H_11,12_); 2.37 (d, *J* = 1.1 Hz, 3H, H_9_); 2.31 (s, 3H, H_13_). ^13^C-NMR (101 MHz, CDCl_3_) δ 162.4 (C_2_); 161.2, 153.2, 152.5, 124.6, 113.3, 112.2 (C_coumarin-aromatic_); 110.6, 107.5 (C_3,4_); 54.7, 53.7 (C_11,12_); 52.5 (C_10_); 45.8 (C_13_); 18.7 (C_9_). LC-MS: rt = 0.9 min, *m/z* 289.33 [M + H]^+^. Purity: 92%, UV (ACN/Water, λ_max_): 322 nm.

*8-(4-Ethylpiperazin-1-ylmethyl)-7-hydroxy-4-methylchromen-2-one* (**4**): FT-IR: (KBr), cm^−1^: 3250–3050 (OH); 3075 (aromatic C–H), 1720 (C=O, lactone), 1600 (C=C, nonaromatic), 1567 (C=C, aromatic). ^1^H-NMR (400 MHz, CDCl_3_) δ 7.41 (d, *J* = 8.7 Hz, 1H, H_5_); 6.76 (d, *J* = 8.7 Hz, 1H, H_6_); 6.07 (q, *J* = 1.1 Hz, 1H, H_3_); 4.07 (s, 2H, H_10_); 3.77–2.50 (m, 8H, H_11,12_); 2.44 (q, *J* = 7.2 Hz, 2H, C_13_); 2.37 (d, *J* = 1.1 Hz, 3H, H_9_); 2.16 (s, 1H, OH); 1.08 (t, *J* = 7.2 Hz, 3H, H_14_). ^13^C-NMR (101 MHz, CDCl_3_) δ 162.5 (C_2_), 161.2, 153.7, 153.3, 152.5, 124.6, 113.4 (C_coumarin-aromatic_); 110.6, 107.5 (C_3,4_); 58.9, 52.6 (C_11,12_); 52.0 (C_10_); 52.0 (C_13_); 18.9 (C_9_); 11.9 (C_14_). LC-MS: rt = 0.9 min, *m/z* 303.38 [M + H]^+^, Purity: 95%, UV (ACN/Water, λ_max_): 322 nm. 

*7-Hydroxy-8-[4-(2-hydroxyethyl)piperazin-1-ylmethyl]-4-methylchromen-2-one* (**5**): FT-IR: (KBr), cm^−1^: 3100–3400 (OH); 3222 (aromatic C–H), 1719 (C=O, lactone), 1597 (C=C, nonaromatic), 1567 (C=C, aromatic). ^1^H-NMR: (400 MHz, CDCl_3_) δ 7.41 (d, *J* = 8.7 Hz, 1H, H_5_); 6.76 (d, *J* = 8.7 Hz, 1H, H_6_); 6.07 (q, *J* = 1.1 Hz, 1H, H_3_); 4.07 (s, 2H, H_10_); 3.63 (t, *J* = 5.3 Hz, 2H, H_14_); 3.58–2.55 (m, 8H, H_11,12_); 2.58 (t, *J* = 5.3 Hz, 2H, H_13_); 2.38 (d, *J* = 1.1 Hz, 3H, H_9_), 2.17 (s, 1H, OH). ^13^C-NMR: (101 MHz, CDCl_3_) δ 162.2 (C_2_); 161.1, 153.2, 152.5, 124.6, 113.3, 112.3 (C_coumarin-aromatic_); 110.7, 107.5 (C_3,4_); 59.0, 57.8 (C_11,12_); 53.7 (C_14_); 52.6 (C_10_); 52.5 (C_13_); 18.8 (C_9_). LC-MS: rt = 0.91 min, *m/z* 319.37 [M + H]^+^, Purity: 94%, UV (ACN/Water, λ _max_): 322 nm Purity: 97%.

*8-(4-Allylpiperazin-1-ylmethyl)-7-hydroxy-4-methylchromen-2-one* (**6**): FT-IR: (KBr), cm^−1^: 3100–3300 (OH); 3067 (aromatic C–H), 3008 (aliphatic C-H); 1728 (C=O, lactone), 1599 (C=C, nonaromatic), 1495 (C=C, aromatic). ^1^H-NMR: (400 MHz, CDCl_3_) δ 7.40 (d, *J* = 8.7 Hz, 1H, H_5_); 6.75 (d, *J* = 8.7 Hz, 1H, H_6_); 6.07 (d, *J* = 1.2 Hz, 1H, H_3_); 5.84 (tt, *J*_1_ = 5.8 Hz, *J*_2_ = 17.0 Hz, H_14_); 5.18 (ddt, *J*_1_ = 1.5 Hz, *J*_2_ = 1.5 Hz, *J_3_* = 17 Hz, 2H, H_15_); 4.06 (s, 2H, H_10_); 3.02 (dt, *J*_1_ = 1.5 Hz, *J*_2_ = 5.8 Hz, 2H, H_13_); 2.98–2.40 (m, 8H, H_11,12_); 2.37 (d, *J* = 1.2 Hz, 3H, H_9_). ^13^C-NMR: (101 MHz, CDCl_3_) δ 162.4 (C_2_); 161.2, 153.2, 152.4, 124.6, 113.3, 112.2 (C_coumarin-aromatic_); 134.4, 118.4 (C_14,15_); 110.6, 107.5 (C_3,4_); 61.4 (C_13_); 53.8, 52.5 (C_11,12_); 52.5 (C_10_); 18.8 (C_9_). LC-MS: rt = 0.91 min, *m/z* 319.37 [M + H]^+^, Purity: 97%, UV (ACN/Water, λ_max_): 322 nm Purity: 95%.

*7-Hydroxy-4-methyl-8-(4-phenylpiperazin-1-ylmethyl)chromen-2-one* (**7**): FT-IR: (KBr), cm^−1^: 3200–3000 (OH); 3047 (aromatic C–H); 2956 (aliphatic C–H); 1715 (C=O, lactone); 1626 (C=C, nonaromatic); 1599 (C=C, aromatic). ^1^H-NMR (400 MHz, CDCl_3_) δ 7.43 (d, *J* = 8.7 Hz, 1H, H_5_); 7.96–7.27 (m, 2H, H_15_); 6.96–6.87 (m, 3H, H_14,16_); 6.79 (d, *J* = 8.7 Hz, 1H, H_6_); 6.09 (q, *J* = 1.10 Hz, 1H, H_3_); 4.13 (s, 2H, H_10_); 3.04 (broad s, 8H, H_11,12_); 2.39 (d, *J* = 1.11 Hz, 3H, H_9_). ^13^C-NMR (400 MHz, CDCl_3_) δ 162.2 (C_2_); 161.1, 153.2, 152.5, 129.1, 124.7, 113.3 (C_Coumarin-aromatic_); 150.7 (C_13_); 120.4 (C_15_); 116.4 (C_16_); 112.3, 110.7 (C_3,4_); 107.5 (C_14_); 53.8 (C_10_); 52.6, 49.1 (C_11,12_); 18.7 (C_9_). LC-MS: rt = 5.63 min, *m/z* 351.37 [M + H]^+^. Purity: 97%, UV (ACN/Water, λ_max_): 322 nm.

*8-[4-(4-Chlorophenyl)piperazin-1-ylmethyl]-7-hydroxy-4-methylchromen-2-one* (**8**): FT-IR: (KBr), cm^−1^: 3300–3050 (OH); 3043 (aromatic C–H); 2966 (aliphatic C–H); 1714 (C=O, lactone); 1625 (C=C, nonaromatic); 1599 (C=C, aromatic). ^1^H-NMR: (400 MHz, CDCl_3_) δ 7.43 (d, *J* = 8.75 Hz, 1H, H_5_); 7.22 (d, *J* = 9.0 Hz, 2H, H_15_); 6.84 (d, *J* = 9.0 Hz, 2H, H_14_); 6.79 (d, *J* = 8.75 Hz, 1H, H_6_); 6.10 (q, *J* = 1.2 Hz, 1H, H_3_); 4.13 (s, 2H, H_10_); 3.36–2.63 (br, 8H, H_11,12_); 2.39 (d, *J* = 1.1 Hz, 3H, H_9_). ^13^C-NMR: (101 MHz, CDCl_3_) δ 162.2(C_2_); 161.1, 153.3, 152.5, 125.2, 117.6, 110.8 (C_coumarin-aromatic_); 149.3, 129.1, 124.8, 113.4, (C_phenyl-arom_); 112.4, 107.4 (C_3,4_); 53.7, 52.4 (C_11,12_); 49.1 (C_10_); 18.8 (C_9_). LC-MS: rt = 6.56 min, *m/z* 385.38 [M + H]^+^, Purity: 96%, UV (ACN/Water, λ_max_): 322 nm.

*8-[(4-(4-Fluorophenyl)piperazin-1-yl)methyl]-7-hydroxy-4-methylchromen-2-one* (**9**): ^1^H-NMR: (400 MHz, CDCl_3_) δ 7.43 (d, *J* = 8.7 Hz, 1H, H_5_); 7.01–6.92 (m, 2H, H_15_); 6.91–6.86 (m, 2H, H_14_); 6.78 (d, *J* = 8.7 Hz, 1H, H_6_); 6.09 (q, *J* = 1.21 Hz, 1H, H_3_); 4.13 (s, 2H, H_10_); 3.29–2.65 (br, 8H, H_11,12_); 2.39 (d, *J* = 1.18 Hz, 3H, H_9_). ^13^C-NMR: (400 MHz, CDCl_3_) δ 162.2 (C_2_); 161.2, 153.3, 14.7, 118.3, 118.2, 115.7, 115.5, 113.4, 112.4 (C_arom_); 110.8, 107.5 (C_3,4_); 53.7, 52.6 (C_11,12_); 50.1(C_10_); 18.8 (C_9_). LC-MS: rt = 6.02 min, *m/z* 369.32 [M + H]^+^, Purity: 98%, UV (ACN/Water, λ_max_): 239 nm, 321 nm.

*7-Hydroxy-8-[4-(bromophenyl)-piperazin-1-ylmethyl]-4-methylchromen-2-one* (**10**): FT-IR: (KBr), cm^−1^: 3350–3100 (OH); 3038 (aromatic C–H); 2965 (aliphatic C–H); 1702 (C=O, lactone); 1625 (C=C, nonaromatic); 1598 (C=C, aromatic). ^1^H-NMR (400 MHz, CDCl_3_) δ 11.58 (s, 1H, OH); 7.41 (d, *J* = 8.8 Hz, 1H, H_5_); 7.32 (d, *J* = 8.8 Hz, 2H, H_15_); 6.77 (d, *J* = 8.8 Hz, 3H, H_6,14_); 6.07 (s, 1H, H_3_); 4.10 (s, 2H, H_10_); 3.21 (broad s, 4H, H_12_); 2.72 (broad s, 4H, H_11_); 2.37 (s, 3H, H_9_). ^13^C-NMR: (400 MHz, CDCl_3_) δ 162.1 (C_2_); 161.0, 153.2, 152.5, 124.8, 113.3, 112.4 (C_Coumarin-aromatic_); 149.7 (C_13_); 131.9 (C_15_); 117.9 (C_14_); 112.5 (C_16_); 110.7, 107.5 (C_3,4_); 53.7, 52.4 (C_11,12_); 48.8 (C_10_); 18.8 (C_9_). LC-MS: rt= 6.60 min, *m/z* 432.24, 433.21 [M + H]^+^, Purity: 96%, UV (ACN/Water, λ_max_): 252 nm.

*7-Hydroxy-4-methyl-8-(4-p-tolyl-piperazin-1-ylmethyl)chromen-2-one* (**11**): FT-IR: (KBr), cm^−1^: 3200–3050 (OH); 3008 (aromatic C–H); 2962 (aliphatic C–H); 1703 (C=O, lactone); 1624 (C=C, nonaromatic); 1599 (C=C, aromatic). ^1^H-NMR: (400 MHz, DMSO) δ 7.54 (d, *J* = 8.8 Hz, 1H, H_5_); 7.00 (d, *J* = 8.7 Hz, 2H, H_15_); 6.82 (d, *J* = 8.7 Hz, 2H, H_14_); 6.78 (d, *J* = 8.7 Hz, 1H, H_6_); 6.12 (q, *J* = 1.2 Hz, 1H, H_3_); 3.92 (s, 2H, H_10_); 3.08 (t, *J* = 4.5 Hz, 4H, H_12_); 2.67 (t, *J* = 4.5 Hz, 4H, H_11_); 2.36 (d, *J* = 1.2 Hz, 3H, H_9_); 2.17 (s, 3H, H_17_). ^13^C-NMR: (400 MHz, DMSO-d_6_) δ 162.2 (C_2_); 160.5, 154.5, 153.2, 149.1, 129.8, 128.3, 116.2, 113.3 (C_aromatics_); 110.1, 108.8 (C_3,4_); 52.5, 49.1 (C_11,12_); 40.5 (C_10_); 20.3 (C_17_); 18.8 (C_9_). LC-MS: rt = 6.37 min, *m/z* 365.22 [M + H]^+^, Purity: 95%, UV (ACN/Water, λ_max_): 322 nm.

*8-[4-(2-Fluorophenyl)piperazin-1-ylmethyl]-7-hydroxy-4-methylchromen-2-one* (**12**): FT-IR: (KBr), cm^−1^: 3300–3150 (OH); 3063 (aromatic C–H); 2986 (aliphatic C-H); 1706 (C=O, lactone); 1625 (C=C, nonaromatic); 1594 (C=C, aromatic). ^1^H-NMR (400 MHz, CDCl_3_) δ 7.41 (d, *J* = 8.78 Hz, 1H, H_5_); 7.06–6.90 (m, 4H, H_14,15,16,17_); 6.77 (d, *J* = 8.79 Hz, 1H, H_6_); 6.07 (q, *J* = 1.22 Hz, 1H, H_3_); 4.11 (s, 2H, H_10_); 3.16 (broad s, 4H, H_11_); 2.82 (broad s, 4H, H_12_); 2.37 (d, *J* = 1.24 Hz, 3H, H_9_). ^13^C-NMR (400 MHz, CDCl_3_) δ 162.2 (C_2_); 161.0, 156.9, 154.4, 124.7, 113.3, 112.3 (C_coumarin-aromatic_); 153.2, 152.5, 139.4, 122.9, 119.0, 116.3 (C_13,14,15,16,17,18_); 110.7, 107.5 (C_3,4_); 53.8, 52.6 (C_11,12_); 50.2 (C_10_); 18.7 (C_9_). LC-MS: rt = 6.17 min, *m/z* 369.19 [M + H]^+^, Purity: 95%, UV (ACN/Water, λ_max_): 322 nm.

*8-(4-Benzo[1,3]dioxol-5-ylmethyl-piperazin-1-ylmethyl)-7-hydroxy-4-methyl-chromen-2-one* (**13**): FT-IR: (KBr), cm^−1^: 3250–3110 (OH); 3047 (aromatic C–H); 2950 (aliphatic C-H); 1718 (C=O, lactone); 1595 (C=C, aromatic). ^1^H-NMR (400 MHz, CDCl_3_) δ 10.52 (s, 1H, OH); 7.37 (d, *J* = 8.7 Hz, 1H), 6.80–6.70 (m, 3H, H_6+15,17_); 6.01 (broad s, 1H, H_18_); 5.90 (q, 1H, *J* = 1.1 Hz, H_3_); 4.02 (s, 2H, H_10_); 3.41 (s, 2H, H_13_); 2.52 (m, 8H, H_11,12_); 2.34 (s, 3H, H_9_). ^13^C-NMR (400 MHz, CDCl_3_) δ 162.4 (C_2_); 161.2, 153.3, 152.4, 124.5, 122.1, 112.1, 110.5 (C_Coumarin-aromatic_); 147.6, 146.6 (C_phenyl_); 131.4 (C_14_); 122.1 (C_18_); 113.3 (C_15_); 109.3 (C_17_); 107.8, 107.5 (C_3,4_); 100.8 (C_16_); 62.3 (C_13_); 53.8, 52.5 (C_11,12_); 52.4 (C_10_); 18.7 (C_9_). LC-MS: rt = 9.81 min, *m/z* 409.38; [M + H]^+^, Purity: 96%, UV (ACN/Water, λ_max_): 220 nm, 321 nm.

### 3.2. Carbonic Anhydrase Inhibition Assay

An Applied Photophysics stopped-flow instrument has been used for assaying the CA catalyzed CO_2_ hydration activity [24]. Phenol red (at a concentration of 0.2 mM) has been used as indicator, working at the absorbance maximum of 557 nm, with 20 mM Tris (pH 8.3) as buffer, and 20 mM Na_2_SO_4_ (for maintaining constant the ionic strength), following the initial rates of the CA-catalyzed CO_2_ hydration reaction for a period of 10–100 s. The CO_2_ concentrations ranged from 1.7 to 17 mM for the determination of the kinetic parameters and inhibition constants. For each inhibitor, at least six traces of the initial 5–10% of the reaction have been used for determining the initial velocity. The uncatalyzed rates were determined in the same manner and subtracted from the total observed rates. Stock solutions of inhibitor (0.1 mM) were prepared in distilled-deionized water and dilutions up to 0.005 nM were done thereafter with the assay buffer. Inhibitor and enzyme solutions were preincubated together for 6 hours at room temperature prior to assay, in order to allow for the formation of the E-I complex. The inhibition constants were obtained by nonlinear least-squares methods using PRISM 3 (GraphPad Software, San Diego, CA, USA) and the Cheng–Prusoff equation, as reported by Maresca et al. [14], and represent the mean from at least three different determinations. All CA isoforms were recombinant ones obtained in-house.

### 3.3. Molecular Modeling

*Molecular docking.* The crystal structures of *h*CA I (PDB code 1AZM), *h*CA II (PDB code 2AW1), *h*CA IX (PDB code 3IAI) and *h*CA XII (PDB code 1JD0) were taken from the Protein Data Bank [25]. Molecular docking calculations were performed with Autodock 4.2 [26] using the improved force field for the treatment of the zinc ion within *h*CAs binding site [27,28]. Autodock Tools were employed for identifying the torsion angles in the ligand, add the solvent model and assign the Kollman atomic charges to the protein. Ligand charges were calculated with the Gasteiger method. A grid spacing of 0.375 Å and a distance-dependent function of the dielectric constant were used for the energetic map calculations. The ligands were subjected to a docking procedure already used in pose prediction studies [29]. The docked compounds were subjected to 100 runs of the Autodock search using the Lamarckian Genetic Algorithm, performing 5,000,000 steps of energy evaluation. All other settings were left as their defaults and the best docked conformation was taken into account for each ligand.

*Molecular dynamics simulations.* Although Autodock scoring function was already successfully used for analyzing the selectivity profile of small-molecule ligands [30], in this case, molecular dynamics (MD) simulations were employed to study the reliability of the predicted ligand binding poses. In fact, the presence of the zinc ion within the protein binding site and the multistep interaction scheme typical of coumarin ligands (binding to the enzyme in both native and hydrolyzed form) would hamper the reliability of ligand binding energy evaluations. Therefore, the ligand–protein complexes generated by docking compound **13** in both native and hydrolyzed form into the catalytic sites of the four *h*CA isoforms were studied through MD simulations with AMBER 16 (University of California, San Francisco, CA, USA) [31]. Each complex was subjected to an MD protocol already applied in pose prediction studies [32,33]. Prior to MD simulations, the system was energy minimized through 5000 steps of steepest descent followed by conjugate gradient until a convergence of 0.05 kcal/(mol·Å^2^) was reached. The minimized complexes were then used as the starting point for three subsequent MD steps, for a total of 30 ns of MD simulation. A 0.5 ns constant-volume simulation in which the temperature of the system was raised from 0 to 300 K was initially performed. In the second step, the system was equilibrated through a 29.5 ns constant-pressure simulation, maintaining the temperature at the constant value of 300 K with the use of Langevin thermostat. All simulations were performed using particle mesh Ewald electrostatics with a cutoff of 10 Å for non-bonded interactions and periodic boundary conditions. A simulation step of 2.0 fs was employed, as all bonds involving hydrogen atoms were kept rigid using the SHAKE algorithm, and General Amber force field (GAFF) parameters were used for the ligand, whose partial charges were calculated with the AM1-BCC method as implemented in the Antechamber suite of AMBER 16.

## 4. Conclusions

A novel series of coumarin derivatives, functionalized with aliphatic and aromatic piperazine fragments in position 8 of the chromen-2-one scaffold, were synthesized and evaluated for their inhibitory activity against *h*CA I, *h*CA II, *h*CA IX and *h*CA XII. All compounds demonstrated the inhibition of the cancer-related *h*CA isoforms IX and XII with *K*_i_ values in the nanomolar range and were shown to be devoid of any inhibitory activity toward the cytosolic *h*CA I and *h*CA II up to 10 µM concentration in the assay system. In particular, single-digit nanomolar *K*_i_ values for *h*CA XII were determined for almost all tested compounds, which were found to be as potent as the reference inhibitor acetazolamide. These results demonstrate that the synthesized compounds represent novel potent and selective non-classic *h*CA IX/XII inhibitors and confirm the importance of the coumarin scaffold for the development of selective CAIs.

Despite the presence of a coumarin core possibly being associated with side pharmacological effects, our ligands should be endowed with a considerably safe profile. In particular, our compounds should be less prone to form epoxide metabolites that are responsible for the hepatotoxic effect of coumarin, which is, however, low (tolerable daily intake of coumarin = 0.1 mg/kg body weight). In fact, the detoxification pathway of coumarin begins with its cytochrome-mediated 7-hydroxylation, followed by urinary excretion either directly or after glucuronidation, a metabolic pathway that determines low susceptibility to the hepatotoxic effect of coumarin in the majority of the human population [34]. Notably, our compounds should be less likely to undergo epoxidation metabolism, as they all present the 7-hydroxyl group that should promote their glucuronidation and excretion. Moreover, our derivatives lack the bulky moiety in position 3 of the bicyclic scaffold that is typical of coumarin-based pharmacologic agents with anticoagulant action, which antagonize the functions of vitamin K. We indeed demonstrated that anticoagulant coumarin derivatives are devoid of *h*CA inhibitory activity [14]. Finally, the peculiar mechanism of action of these ligands, requiring an in situ CA-catalyzed hydrolysis for generating the cinnamic acids that constitute the real CAIs should prevent any off-target effect that might be caused by their interaction with other zinc-dependent enzymes, such as matrix metalloproteinases.

Our molecular modeling studies addressed the ligand recognition process of the intact coumarin compounds at the level of *h*CAs catalytic site, as well as the binding interactions of the corresponding cinnamic acids produced by CA-mediated hydrolysis, according to mechanism of inhibition typical of coumarin-based ligands. The results suggested that the selectivity of this series of compounds should be ascribed to the ligand recognition process and the interactions of the coumarin scaffold. Moreover, consistently with the experimental evidence, the piperazine-based substituents of the ligands were shown to provide a modest contribution to the ligand–protein interactions predicted for the compounds in both their coumarin and cinnamic form. Our analysis could thus be used to guide lead optimization studies aimed at introducing modified structural moieties for either further improving the ligand recognition process of the coumarin ligands or maximizing the ligand–protein interactions that can be stablished by the hydrolyzed compounds.

## Figures and Tables

**Table 1:** Inhibition data of *h*CA I, *h*CA II, *h*CA IX, *h*CA XII with compounds here reported and the standard sulfonamide inhibitor acetazolamide (**AAZ**) by a stopped flow CO_2_ hydrase assay.

Compound	K_i_ (nM)			
	hCA I	hCA II	hCA IX	hCA XII
**1**	>10,000	>10,000	32.1	5.8
**2**	>10,000	>10,000	67.5	8.5
**3**	>10,000	>10,000	88.7	9.6
**4**	>10,000	>10,000	32.0	6.4
**5**	>10,000	>10,000	294.9	7.1
**6**	>10,000	>10,000	27.1	5.6
**7**	>10,000	>10,000	114.7	7.7
**8**	>10,000	>10,000	190.3	8.9
**9**	>10,000	>10,000	92.8	8.1
**10**	>10,000	>10,000	162.3	9.4
**11**	>10,000	>10,000	307.7	9.6
**12**	>10,000	>10,000	120.7	26.4
**13**	>10,000	>10,000	76.8	8.5
**AAZ**	250	12	25	5.7

* Mean from three different assays, by a stopped flow technique (errors were in the range of ±5–10% of the reported values).

## Supplementary Materials

Supplementary materials can be found at https://www.mdpi.com/1422-0067/20/5/1208/s1.
