# Supplementary material for: Novel 8-Substituted Coumarins That Selectively Inhibit Human Carbonic Anhydrase IX and XII
Source: Int J Mol Sci. 2019 Mar 10;20(5):1208. doi: 10.3390/ijms20051208 (PMC6429297; doi:10.3390/ijms20051208)

Supporting Information

Novel 8-Substituted Coumarins That Selectively Inhibit Human Carbonic Anhydrase IX and XII

Kerem Buran, Silvia Bua, Giulio Poli, F. Esra Önen Bayram, Tiziano Tuccinardi, Claudiu T. Supuran

Table of Contents

Table S1. Yields obtained for compounds 2- 13 S2

Figure S1. Alignment between *h*CA I, *h*CA II, *h*CA IX and *h*CA XII S3

NMR spectra of final compounds. S4-S28

Table S1. Yields obtained for compounds **2- 13**

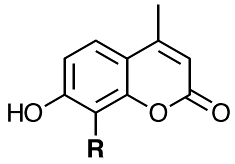

	R Groups	% Yield
**2**	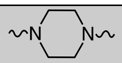	63
**3**	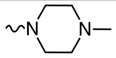	32
**4**	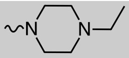	47
**5**	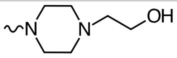	26
**6**	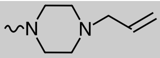	50
**7**	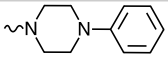	53
**8**	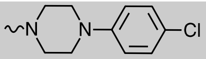	64
**9**	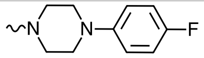	67
**10**	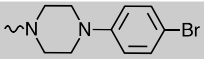	50
**11**	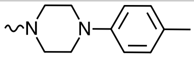	60
**12**	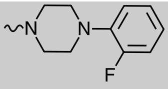	76
**13**	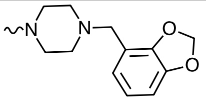	60

CAI --
CAII --
CAXII --
CAIX MAPLCPSPWLPLLIPAPAPGLTVQLLLSLLLLVPVHPQRLPRMQEDSPLG

CAI --
CAII --
CAXII --MP
CAIX GGSSGEDDPLGEEDLPSEEDSPREEDPPGEEDLPGEEDLPGEEDLPEVKP

CAI --------------------------------MASPDWGYDDKN**G**PEQ**W**S
CAII ---------------------------------MSHHWGYGKHN**G**PEH**W**H
CAXII RRSLHAAAVLLLVILKEQPSSPAP--------VNGSKWTYFGPD**G**ENS**W**S
CAIX KSEEEGSLKLEDLPTVEAPGDPQEPQNNAHRDKEGDDQSHWRYG**G**DPP**W**P

CAI KLY**P**IAN**G**NN**QSP**V**D**IKTSETKHDTS**L**K**P**ISVS-YNPATAKEIINVGHSF
CAII KDF**P**IAK**G**ER**QSP**V**D**IDTHTAKYDPS**L**K**P**LSVS-YDQATSLRILNNGHAF
CAXII KKY**P**SCG**G**LL**QSP**I**D**LHSDILQYDAS**L**T**P**LEFQGYNLSANKQFLLTNNGH
CAIX RVS**P**ACA**G**RF**QSP**V**D**IRPQLAAFCPA**L**R**P**LELLGFQLPPLPELRLRNNGH

CAI H**V**NFEDNDNRSVLKGGPFSDS**Y**RLF**Q**F**H**F**HWG**STNE-H**GSEHTV**DGVKYS
CAII N**V**EFDDSQDKAVLKGGPLDGT**Y**RLI**Q**F**H**F**HWG**SLDG-Q**GSEHTV**DKKKYA
CAXII S**V**KLNLPSDMHIQG---LQSR**Y**SAT**Q**L**H**L**HWG**NPNDPH**GSEHTV**SGQHFA
CAIX S**V**QLTLPPGLEMALG--PGRE**Y**RAL**Q**L**H**L**HWG**AAGR-P**GSEHTV**EGHRFP

CAI **AE**L**H**VA**H**WNSAKYSSLAE**A**ASKAD**GLAV**IGVLMKV**G**-EANPKLQKVLDA**L**
CAII **AE**L**H**LV**H**WN-TKYGDFGK**A**VQQPD**GLAV**LGIFLKV**G**-SAKPGLQKVVDV**L**
CAXII **AE**L**H**IV**H**YNSDLYPDAST**A**SNKSE**GLAV**LAVLIEM**G**-SFNPSYDKIFSH**L**
CAIX **AE**I**H**VV**H**LS-TAFARVDE**A**LGRPG**GLAV**LAAFLEE**G**PEENSAYEQLLSR**L**

CAI QAIKTK**G**KRAPFTNFDPST**LLP**S-SLDFWT**Y**P**GSLT**H**PP**LYES**V**T**W**IICK
CAII DSIKTK**G**KSADFTNFDPRG**LLP**E-SLDYWT**Y**P**GSLT**T**PP**LLEC**V**T**W**IVLK
CAXII QHVKYK**G**QEAFVPGFNIEE**LLP**ERTAEYYR**Y**R**GSLT**T**PP**CNPT**V**L**W**TVFR
CAIX EEIAEE**G**SETQVPGLDISA**LLP**SDFSRYFQ**Y**E**GSLT**T**PP**CAQG**V**I**W**TVFN

CAI ESISV**S**SE**Q**LAQFRSLLSNVEGDNAV--PMQH**N**N**R**PT**Q**PLKG**R**TVRA**SF**-
CAII EPISV**S**SE**Q**VLKFRKLNFNGEGEPEE--LMVD**N**W**R**PA**Q**PLKN**R**QIKA**SF**K
CAXII NPVQI**S**QE**Q**LLALETALYCTHMDDPSPREMIN**N**F**R**QV**Q**KFDE**R**LVYT**SF**S
CAIX QTVML**S**AK**Q**LHTLSDTLWG-----PGDSRLQL**N**F**R**AT**Q**PLNG**R**VIEA**SF**P

CAI --
CAII --
CAXII Q-VQVCTAAGLSLGIILSLALAGILGICI-----VVVVSIWLFRRKSIKK
CAIX AGVDSSPRAAEPVQLNSCLAAGDILALVFGLLFAVTSVAFLVQMRRQHRR

CAI --------------------
CAII --------------------
CAXII GDNKGVIYKPATKMETEAHA
CAIX GTKGGVSYRPAEVAETGA--

Figure S1. Alignment between *h*CA I, *h*CA II, *h*CA IX and *h*CA XII. Conserved residues are reported in bold.

Compound 1
^{1}H NMR

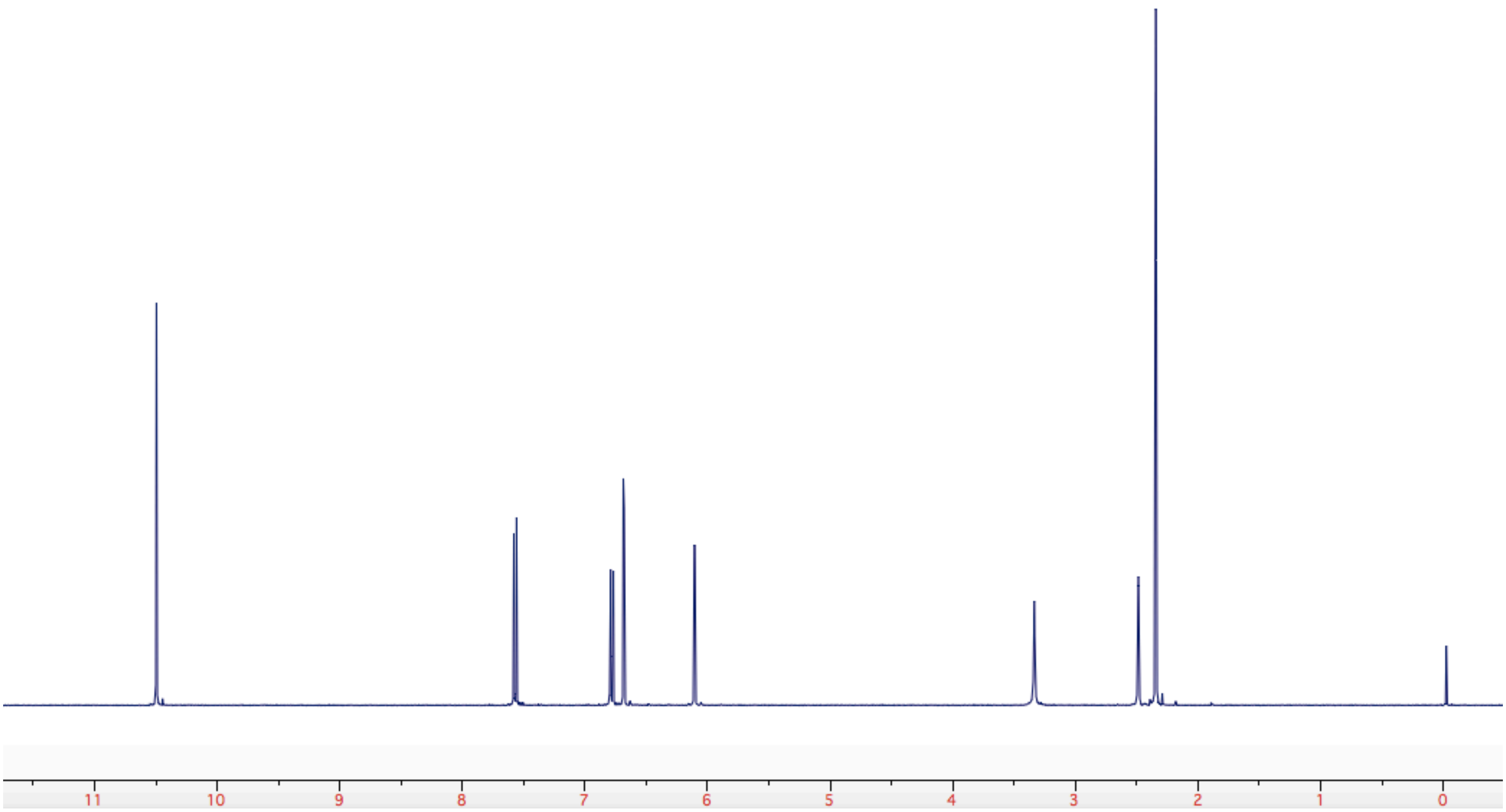

Compound 1
^{13}C NMR

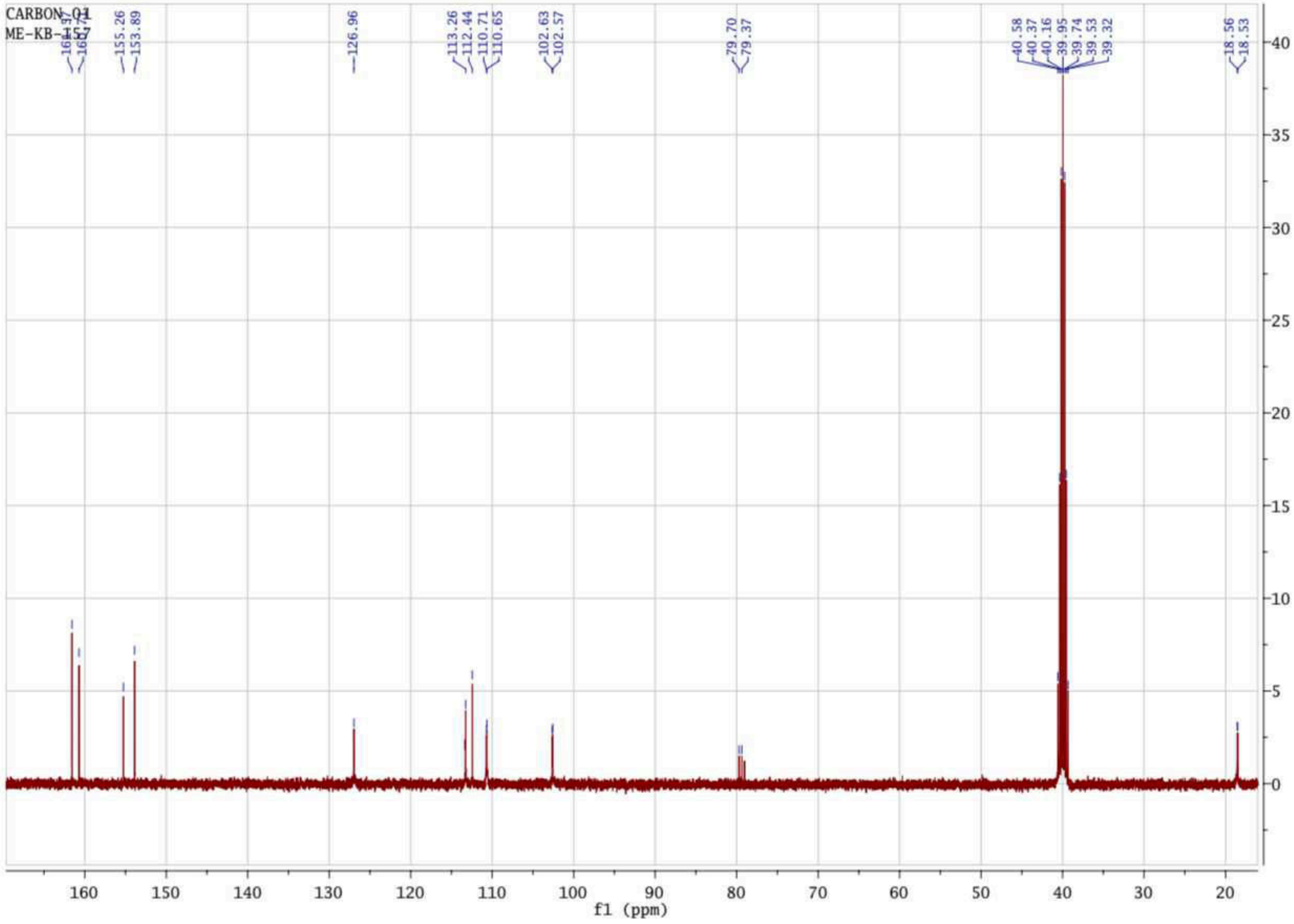

Compound 2

^{1}H NMR

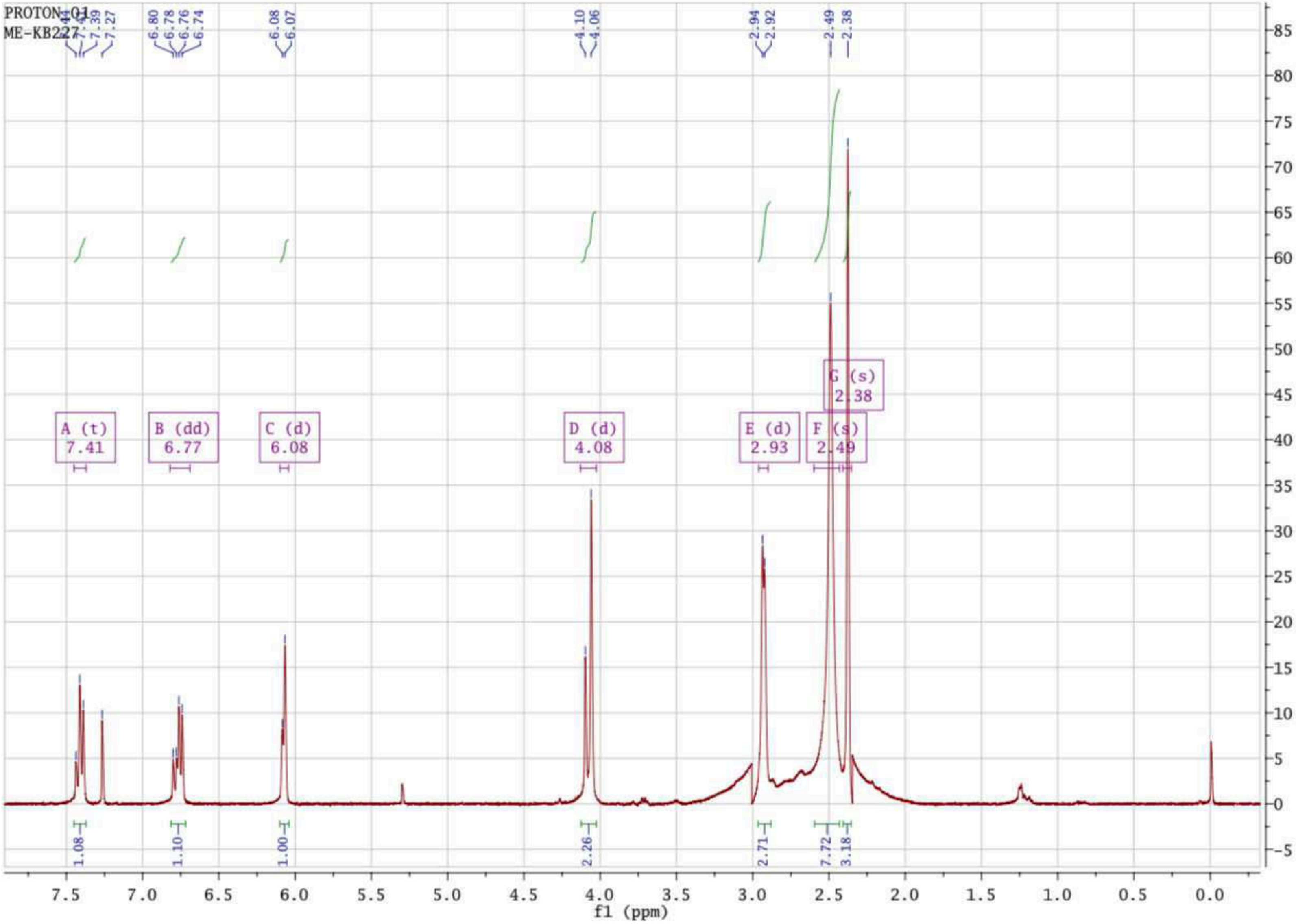

Compound 2
^{13}C NMR

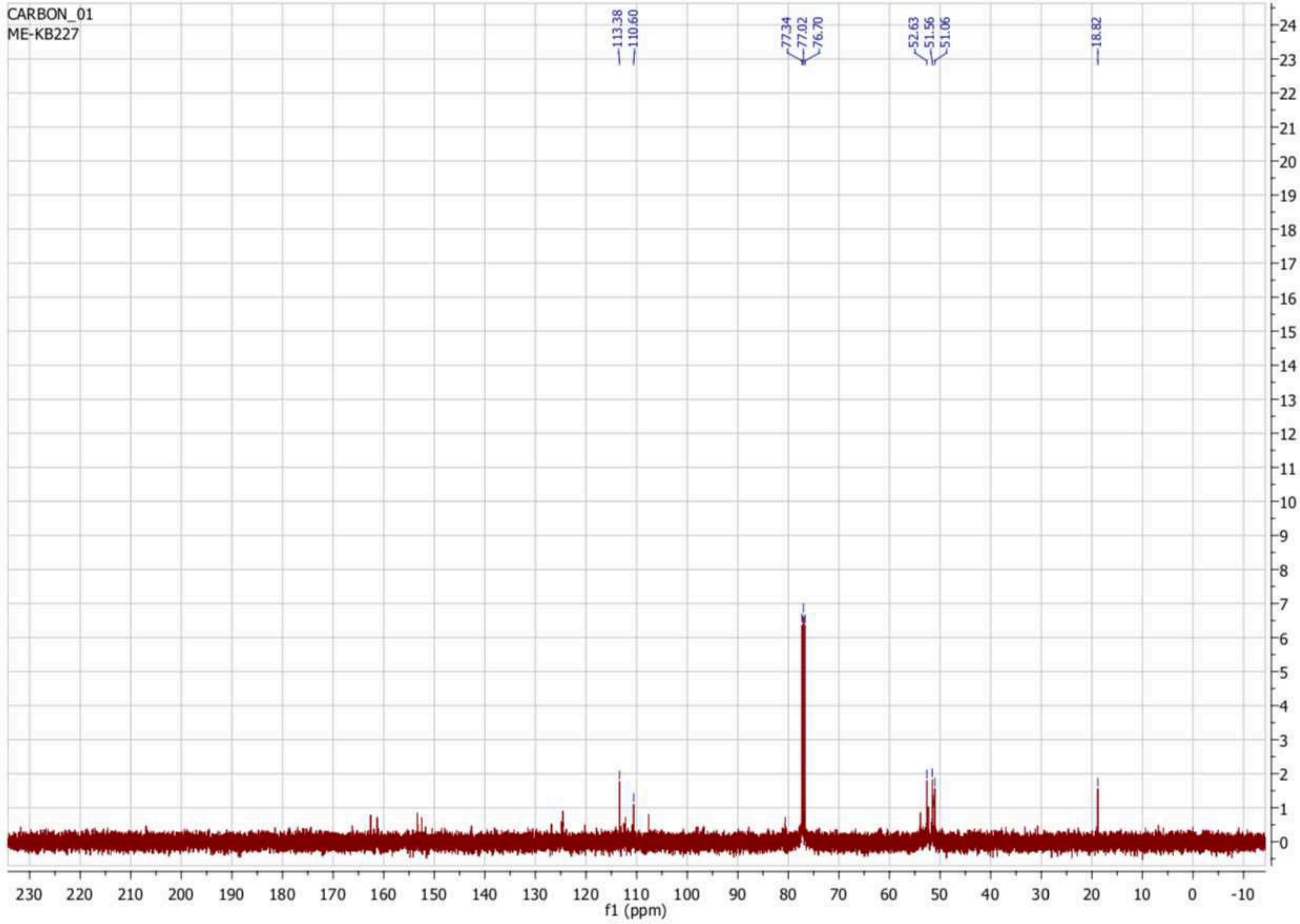

Compound 3
^{1}H NMR

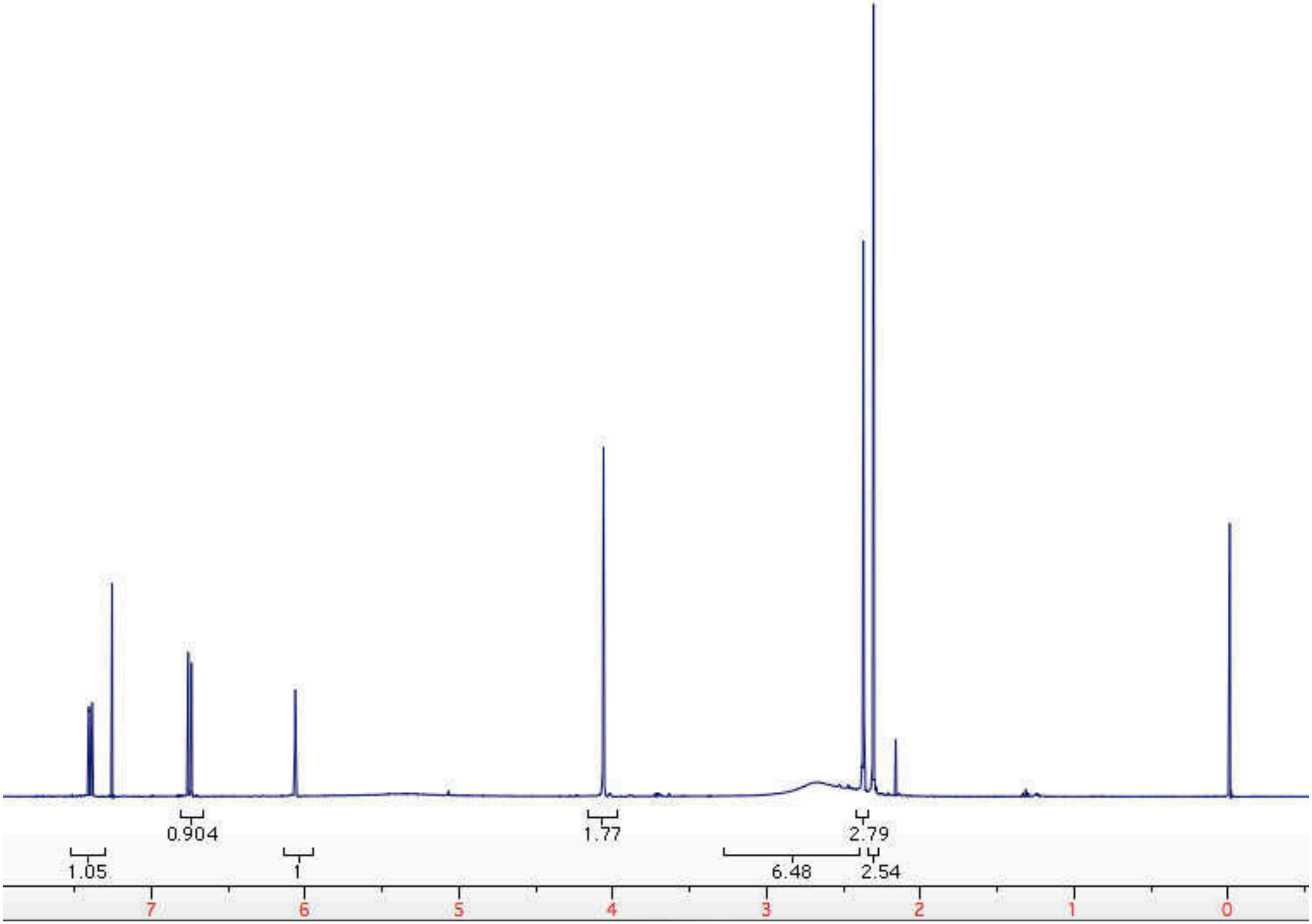

Compound 3
^{13}C NMR

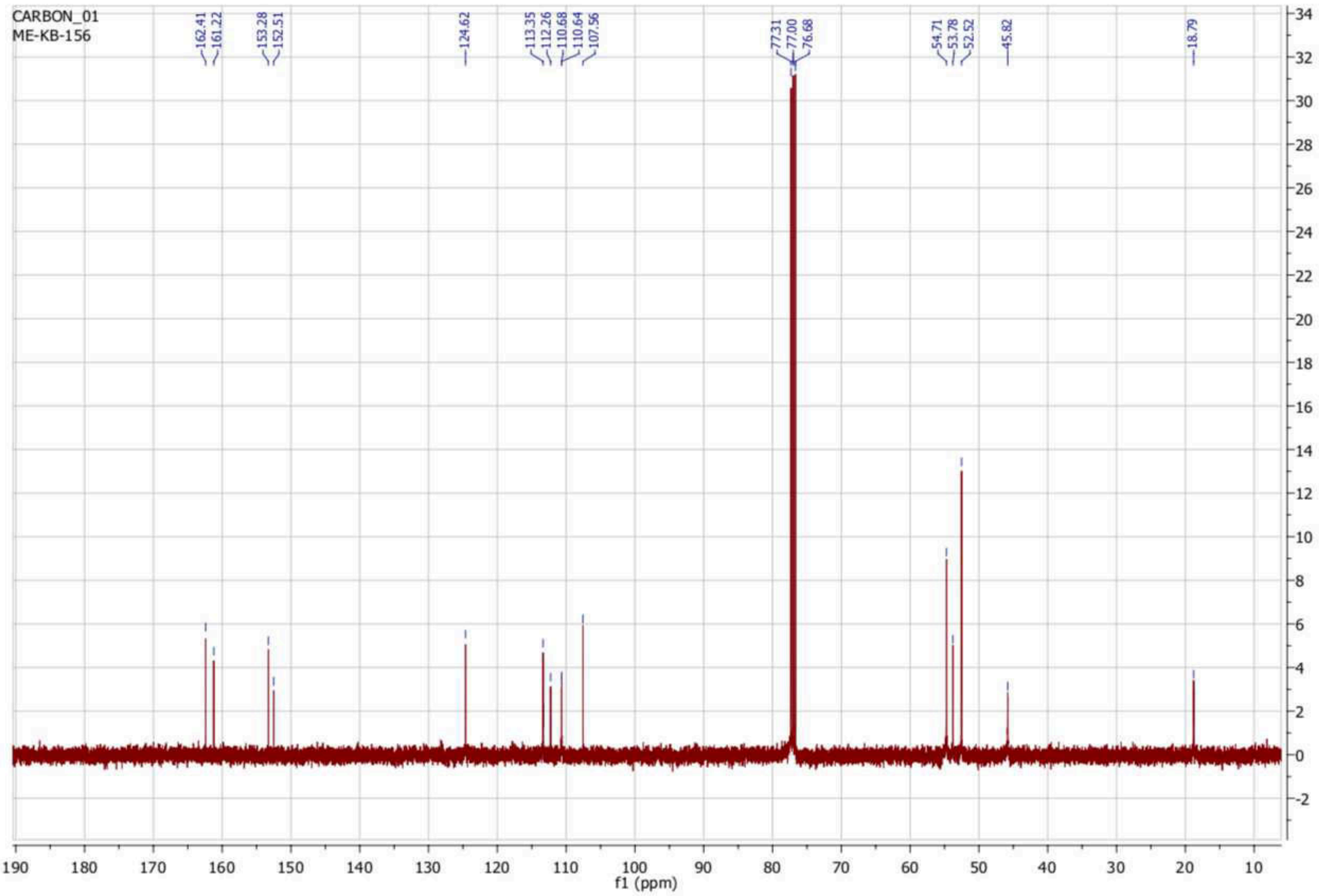

Compound 4
^{1}H NMR

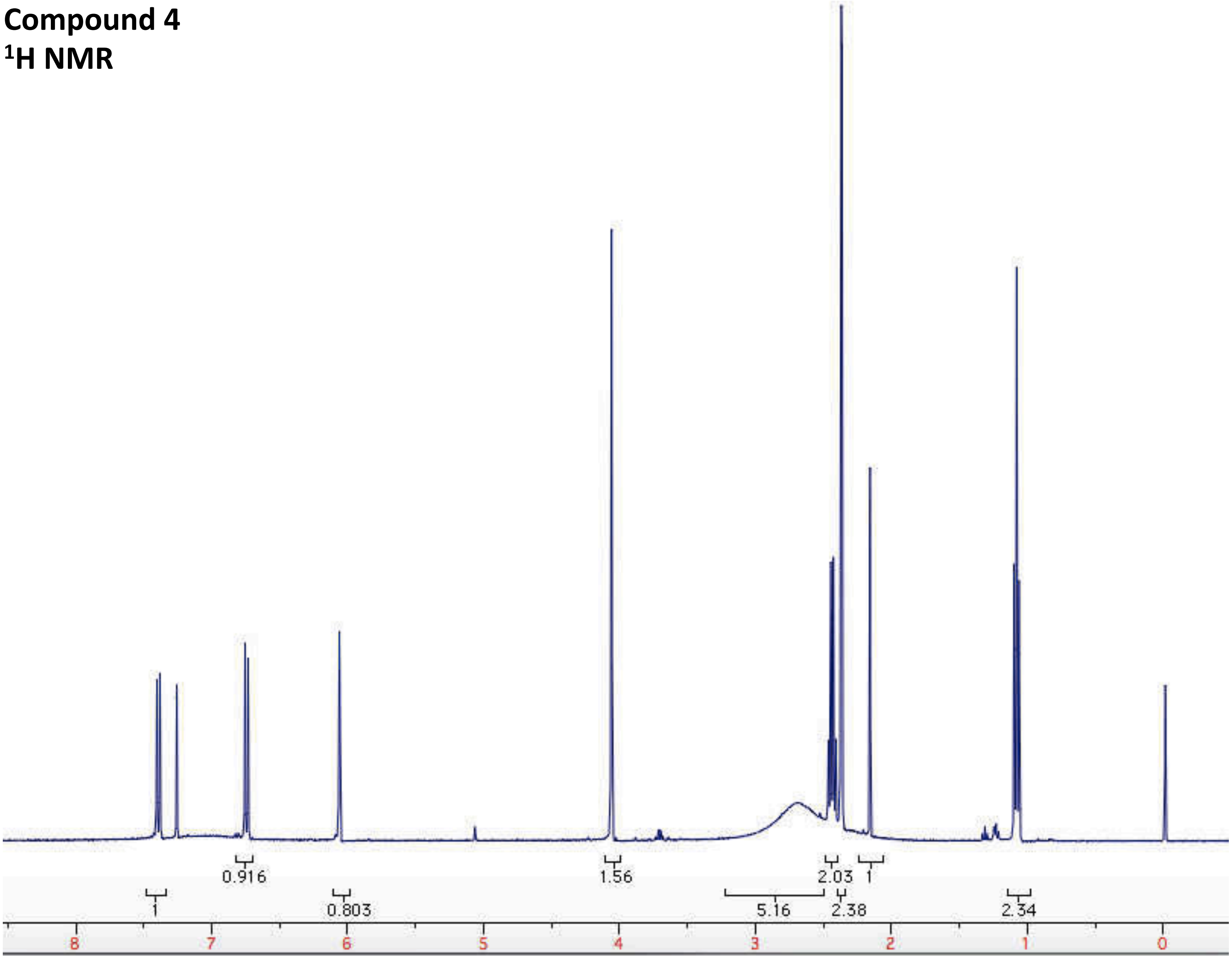

Compound 4
^{13}C NMR

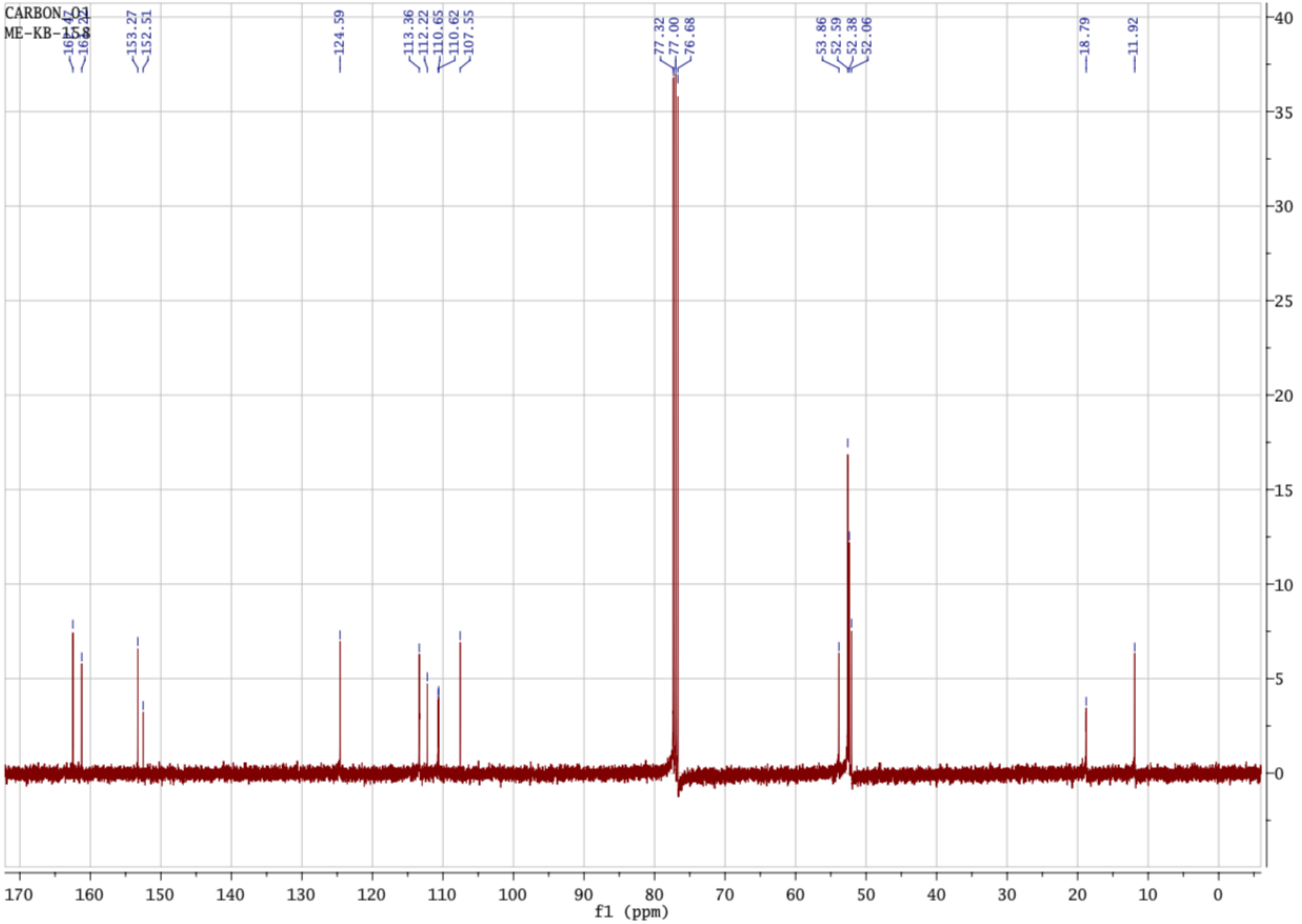

Compound 5
^{1}H NMR

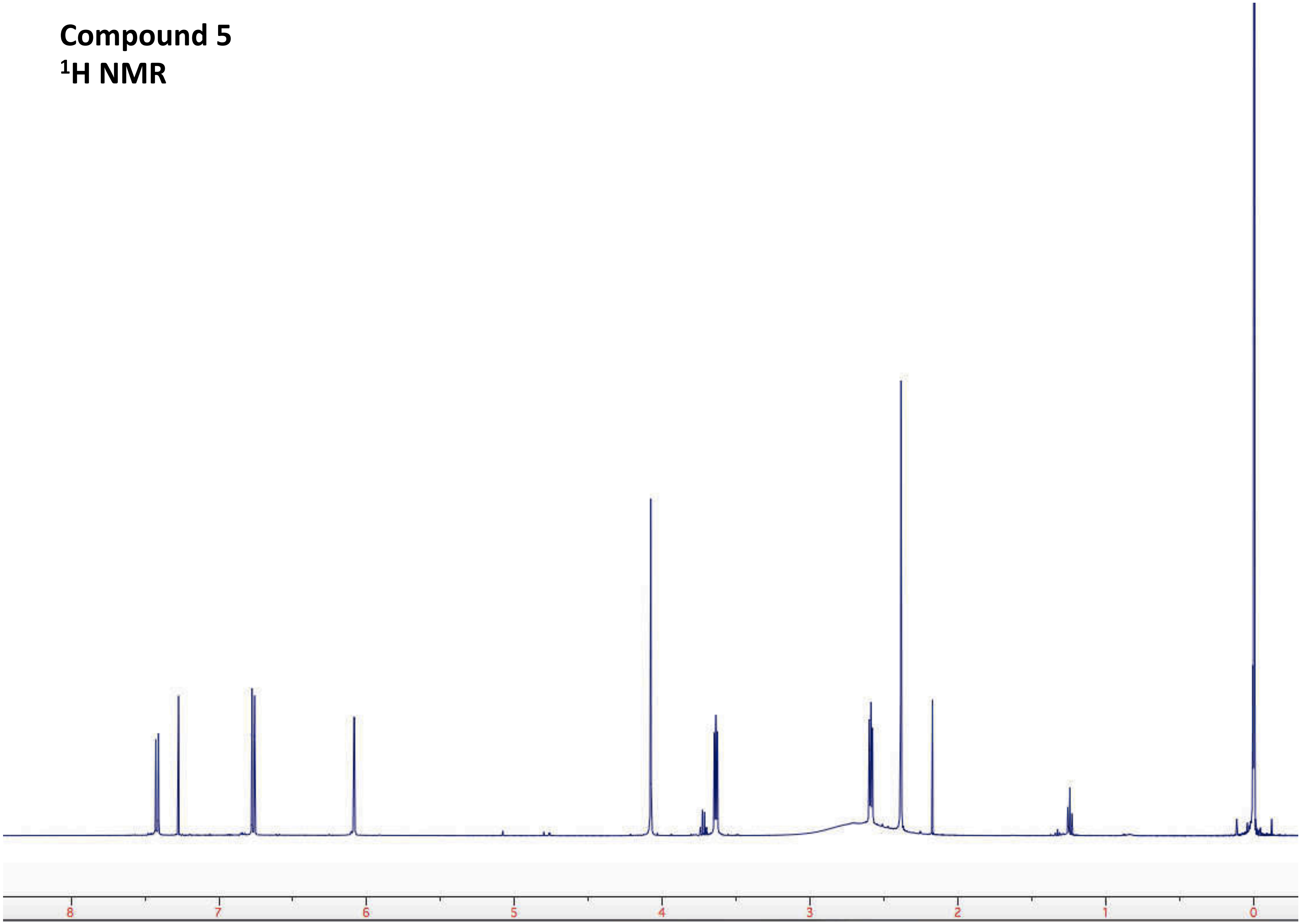

Compound 5
^{13}C NMR

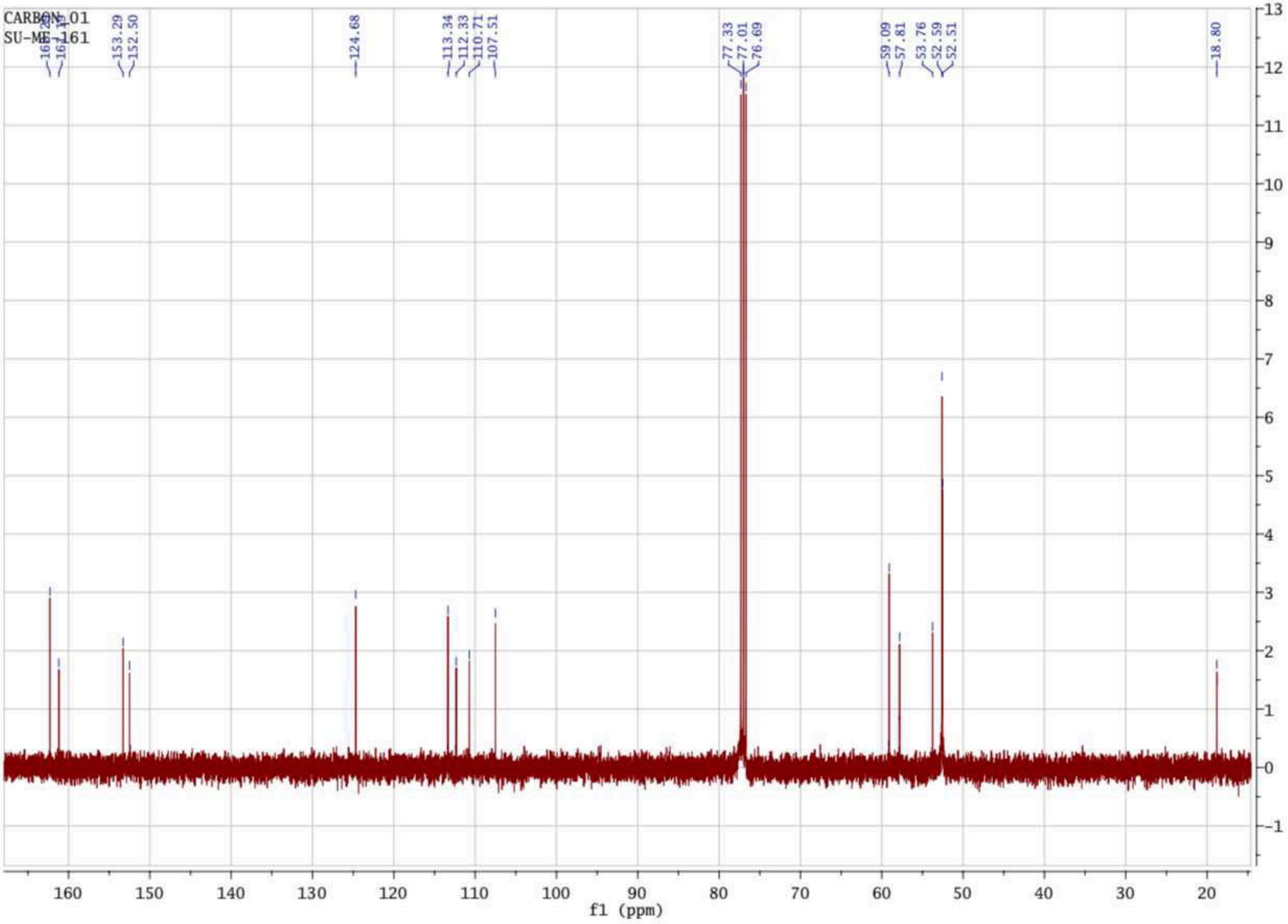

Compound 6
^{1}H NMR

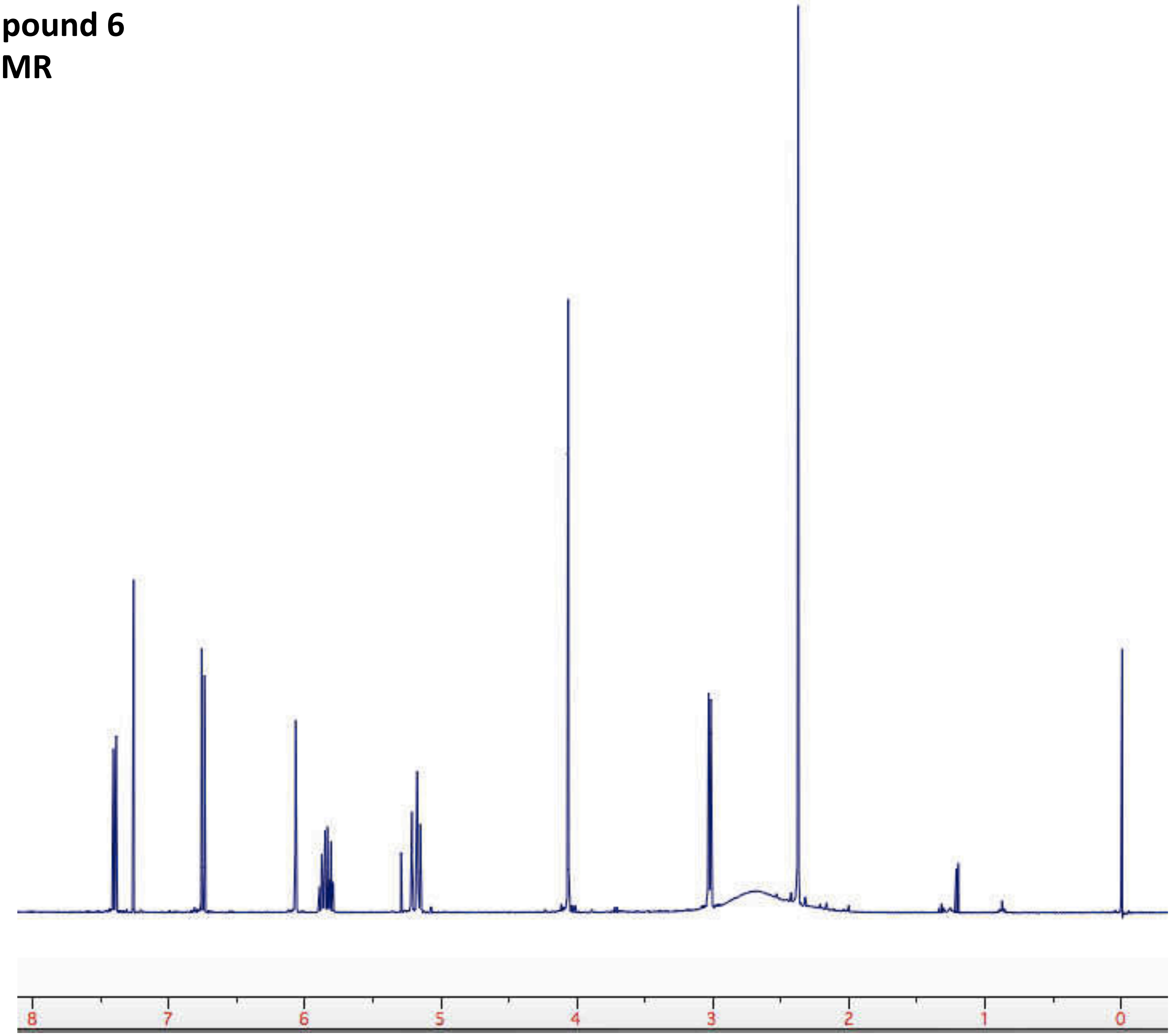

Compound 6
^{13}C NMR

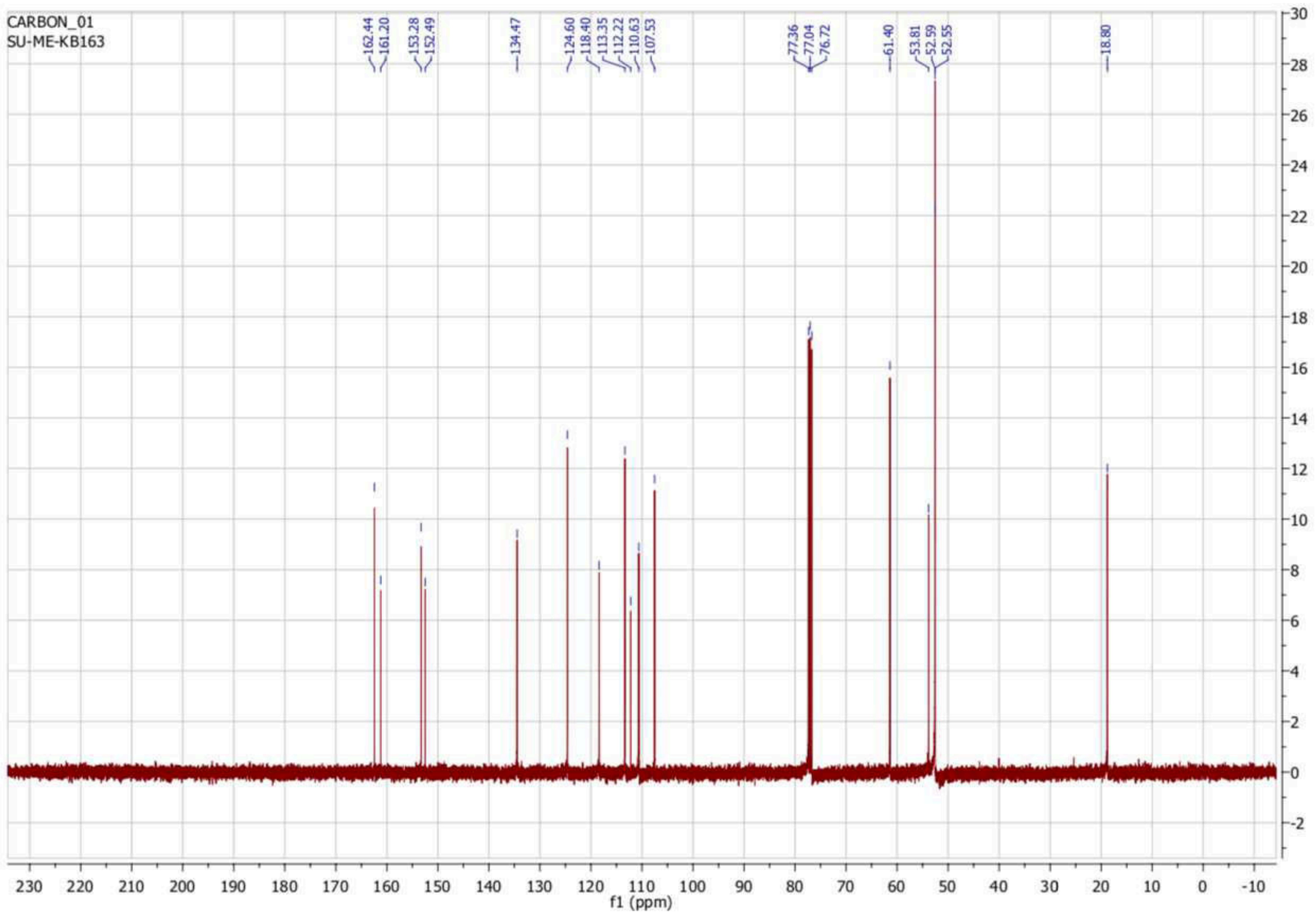

Compound 7
^{1}H NMR

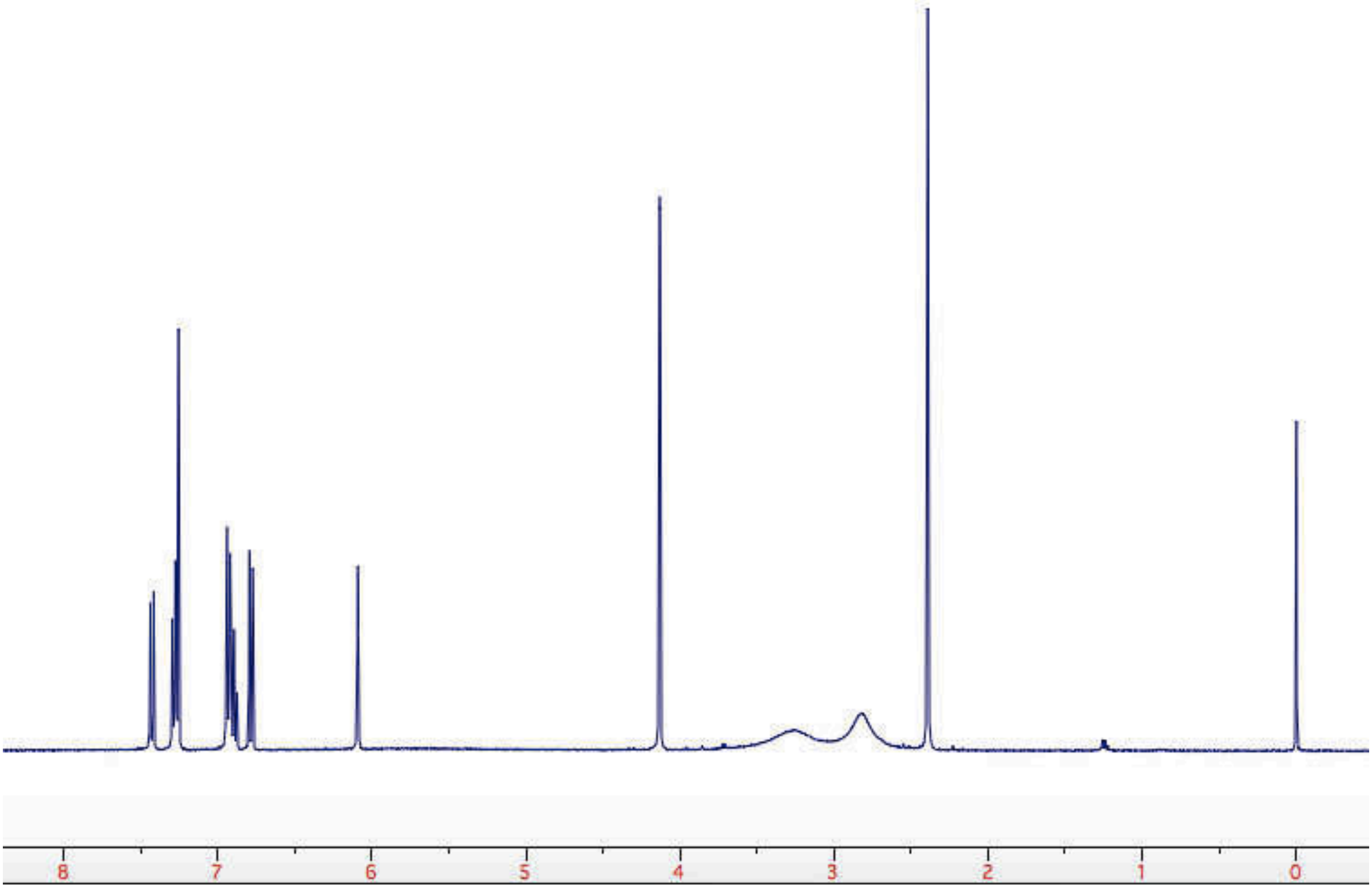

Compound 7
^{13}C NMR

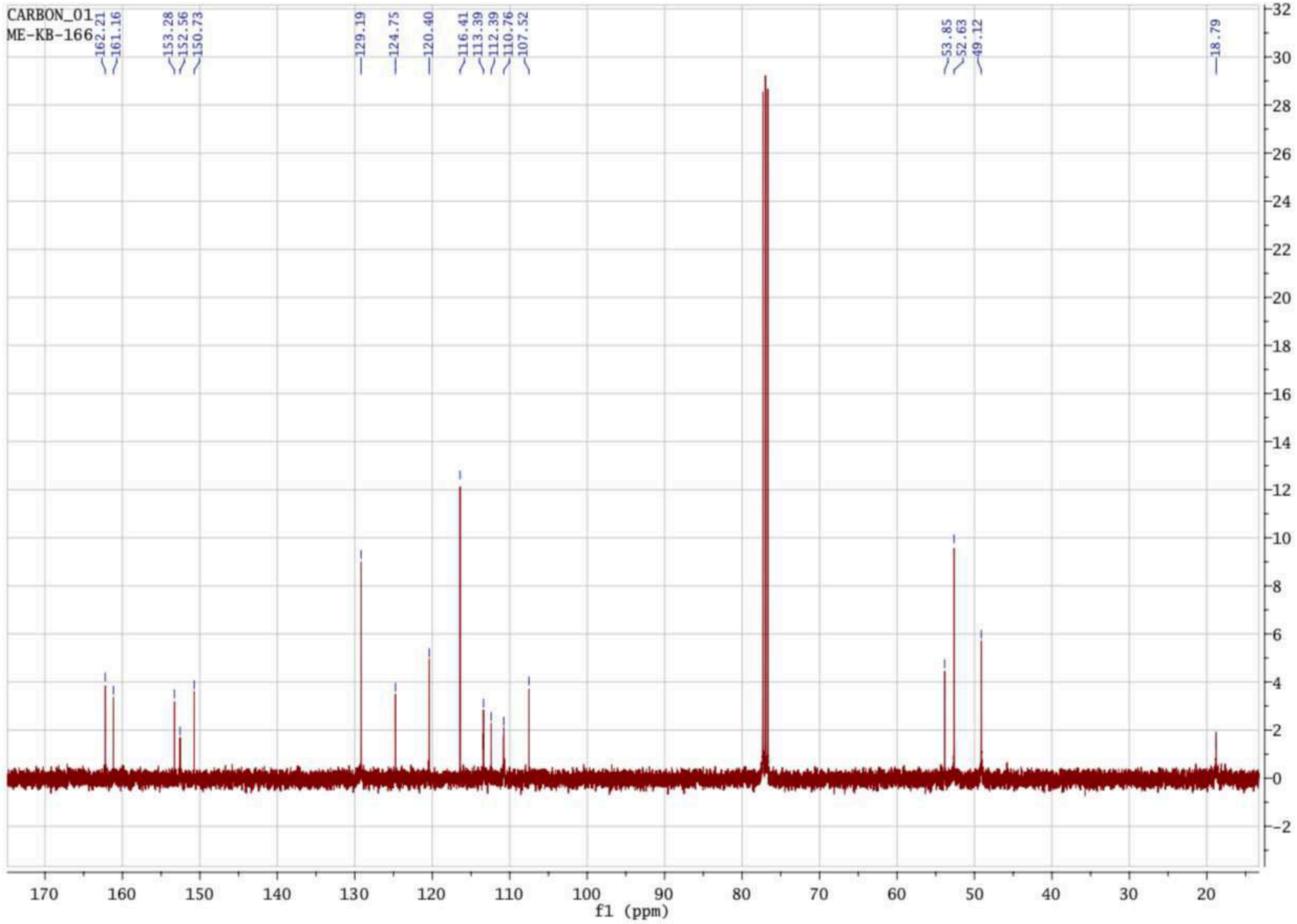

Compound 8
^{1}H NMR

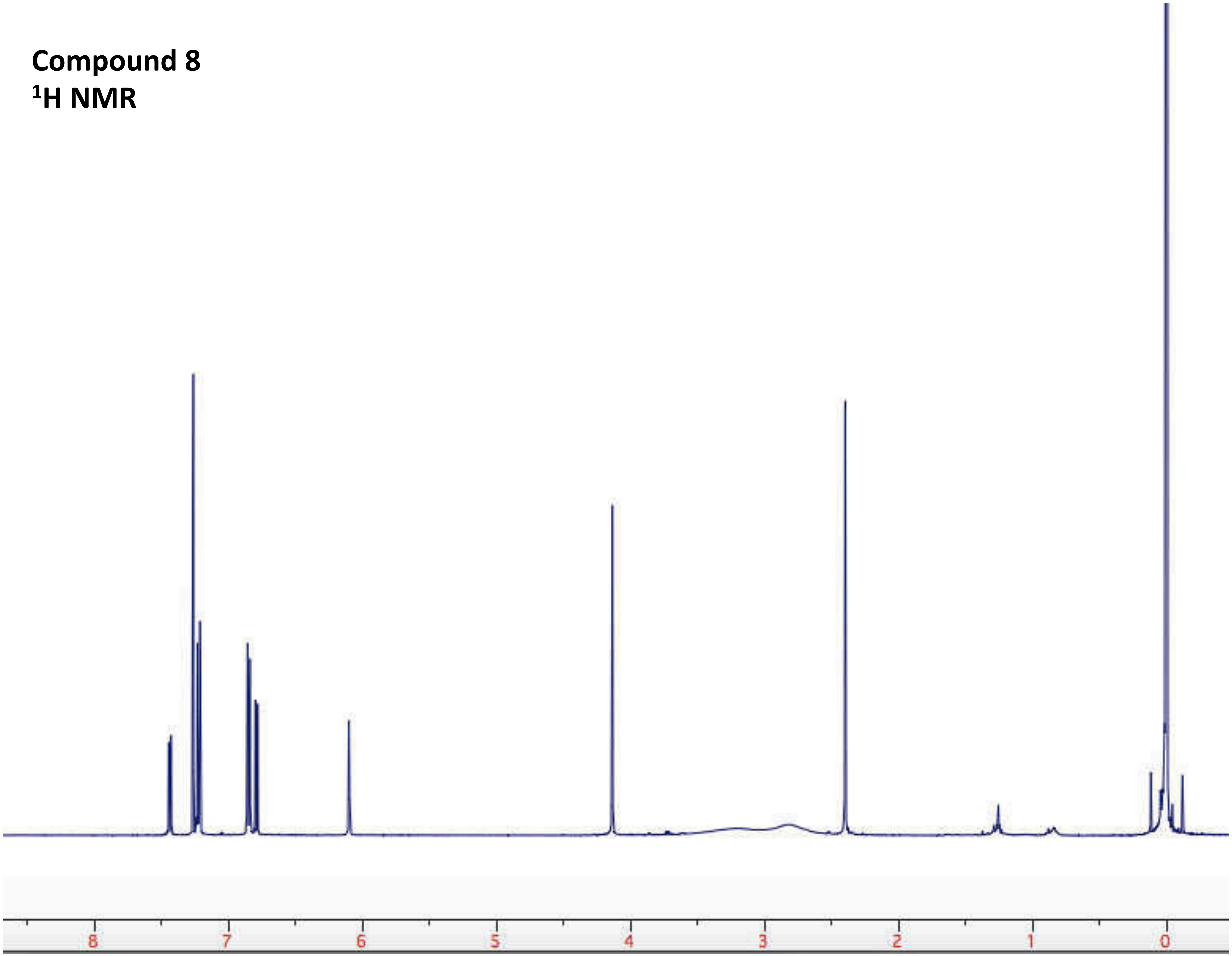

Compound 8
^{13}C NMR

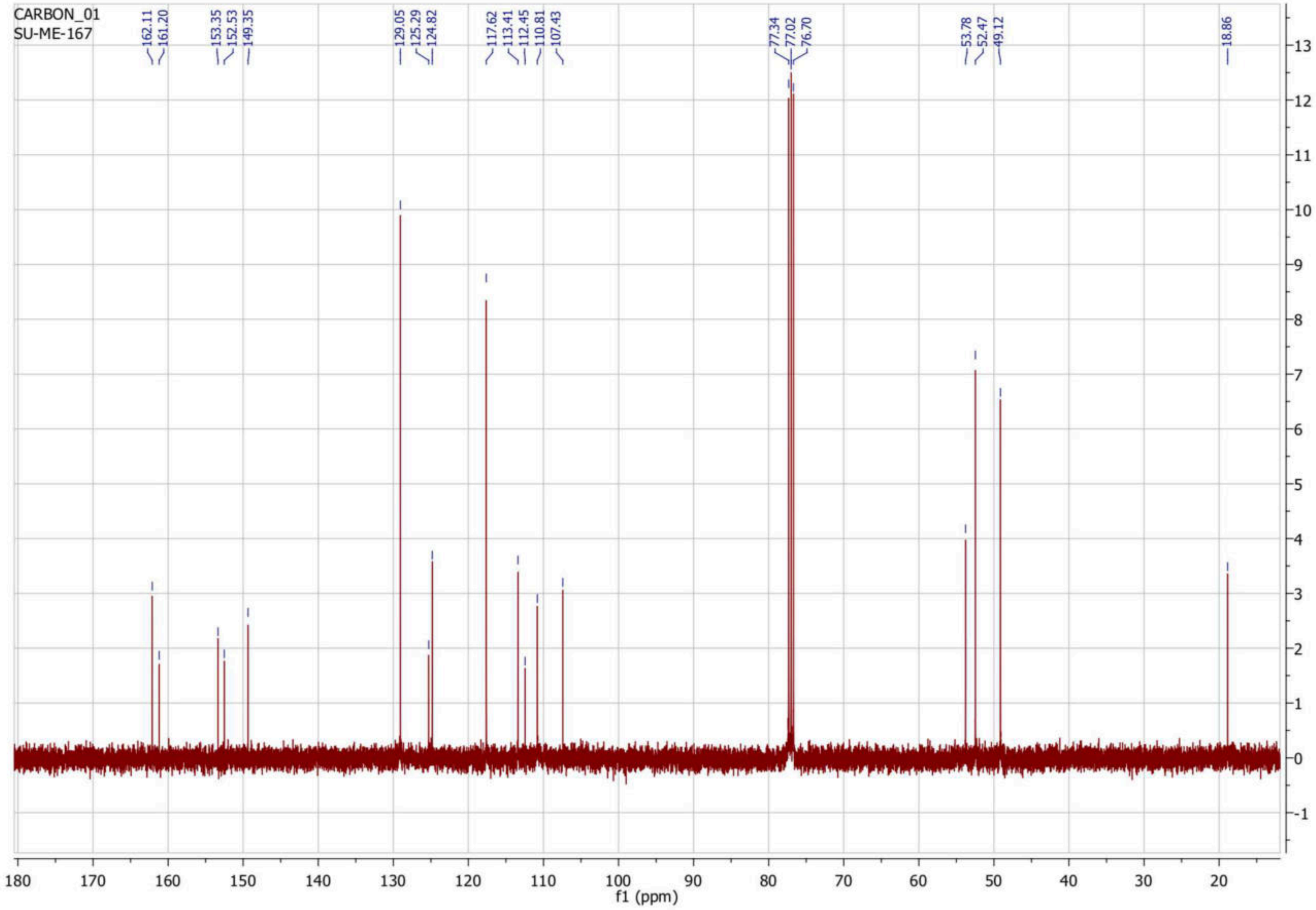

Compound 9
^{1}H NMR

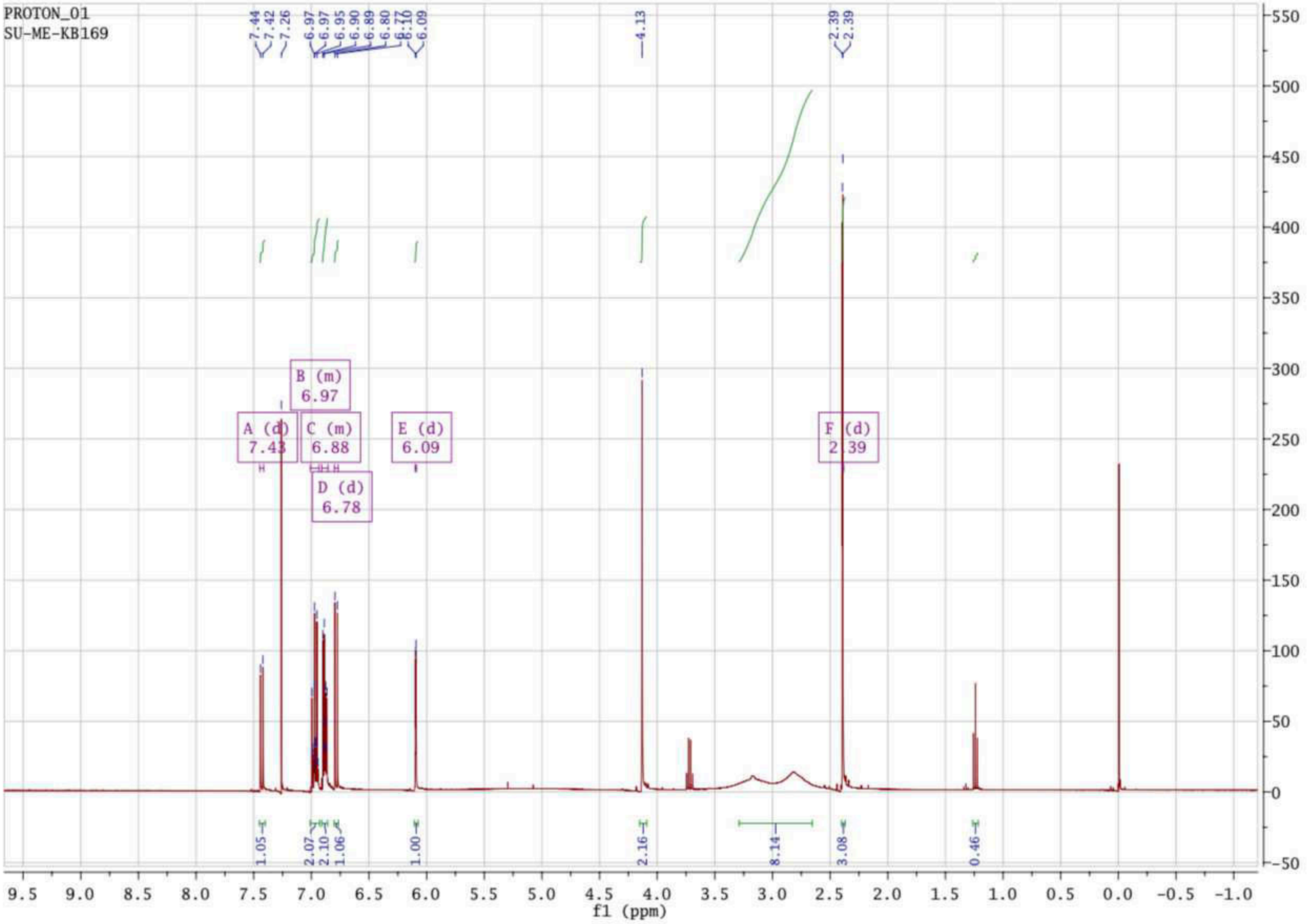

Compound 9
^{13}C NMR

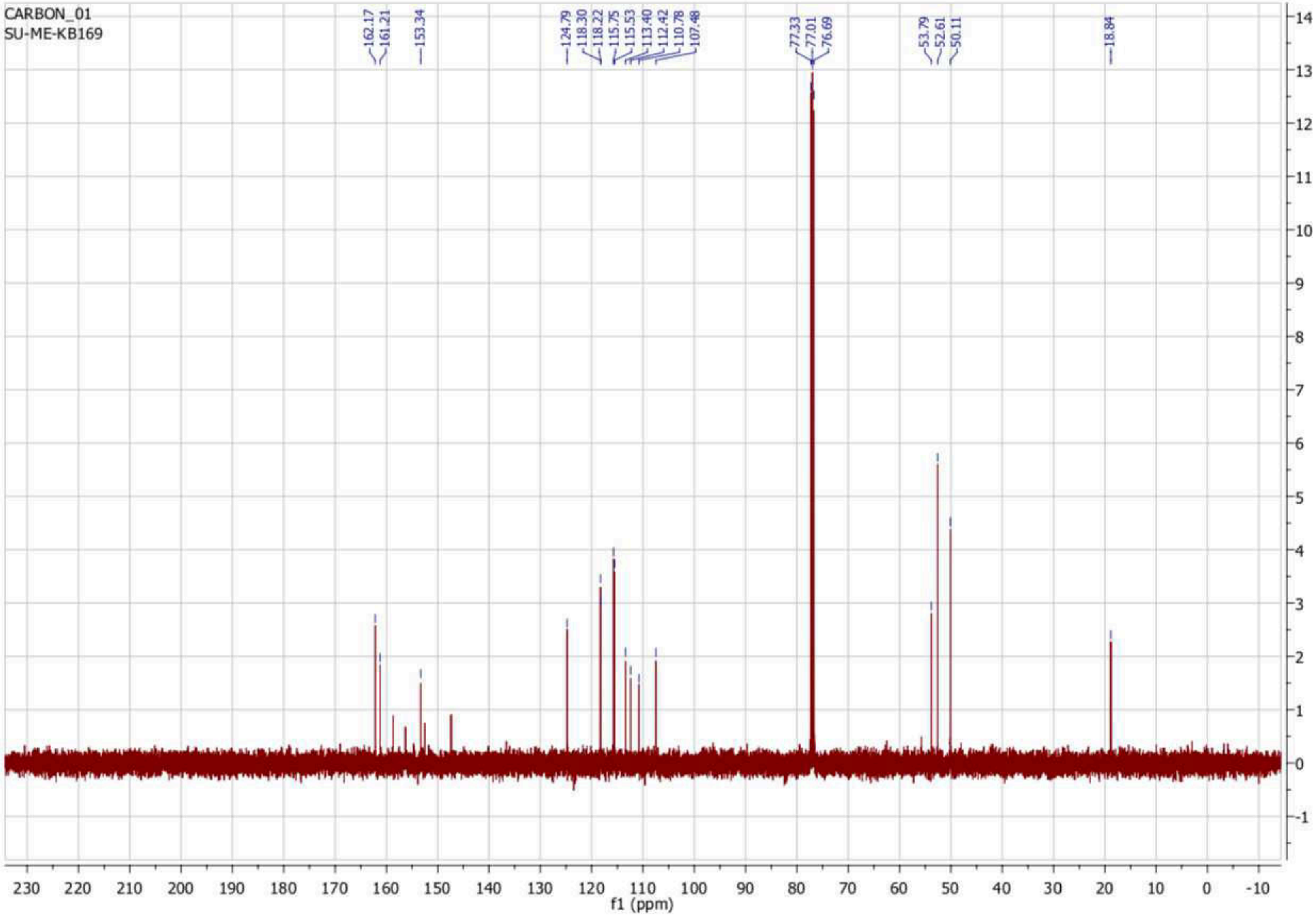

Compound 10
^{1}H NMR

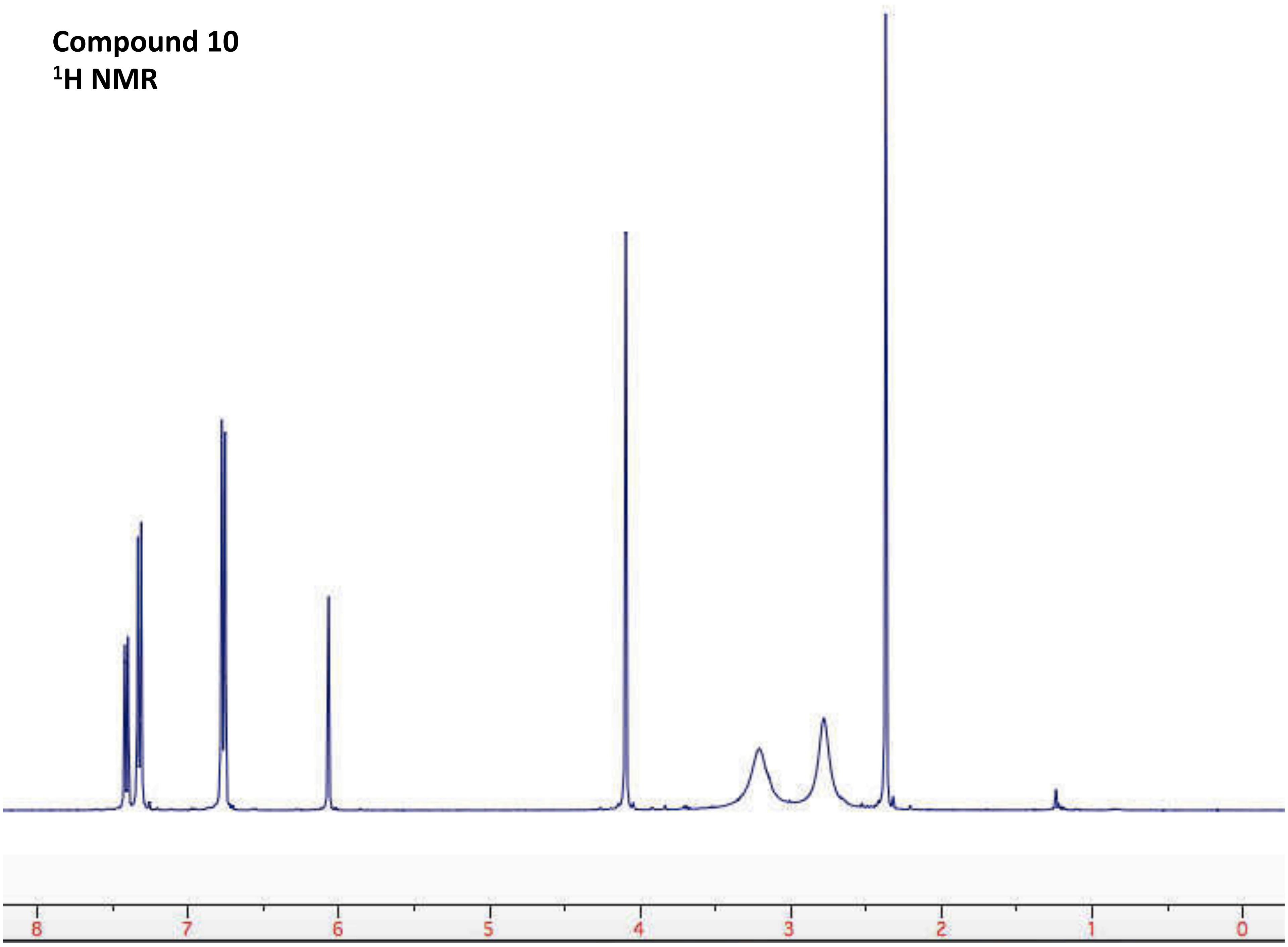

Compound 10
^{13}C NMR

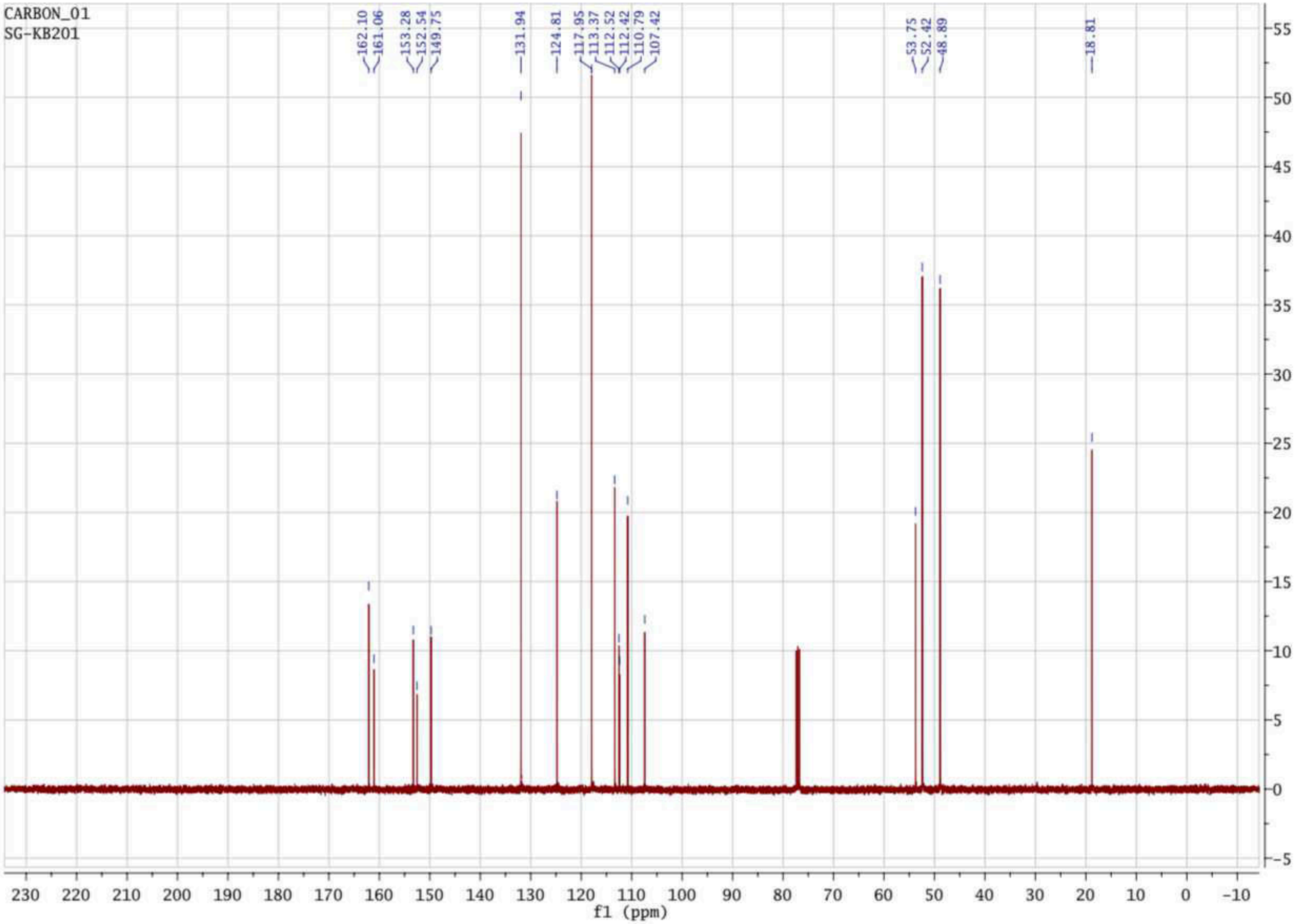

Compound 11
^{1}H NMR

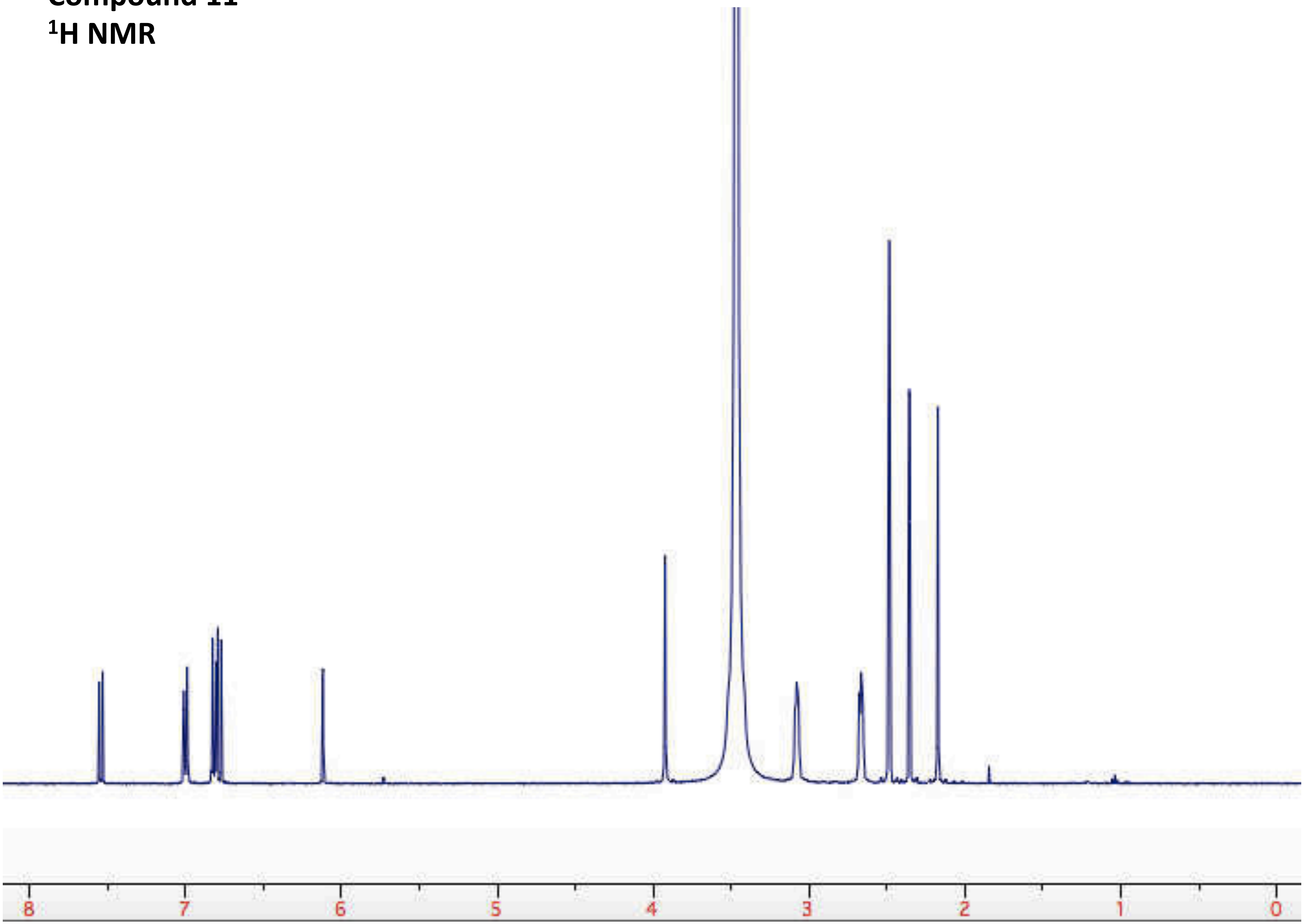

Compound 11

^{13}C NMR

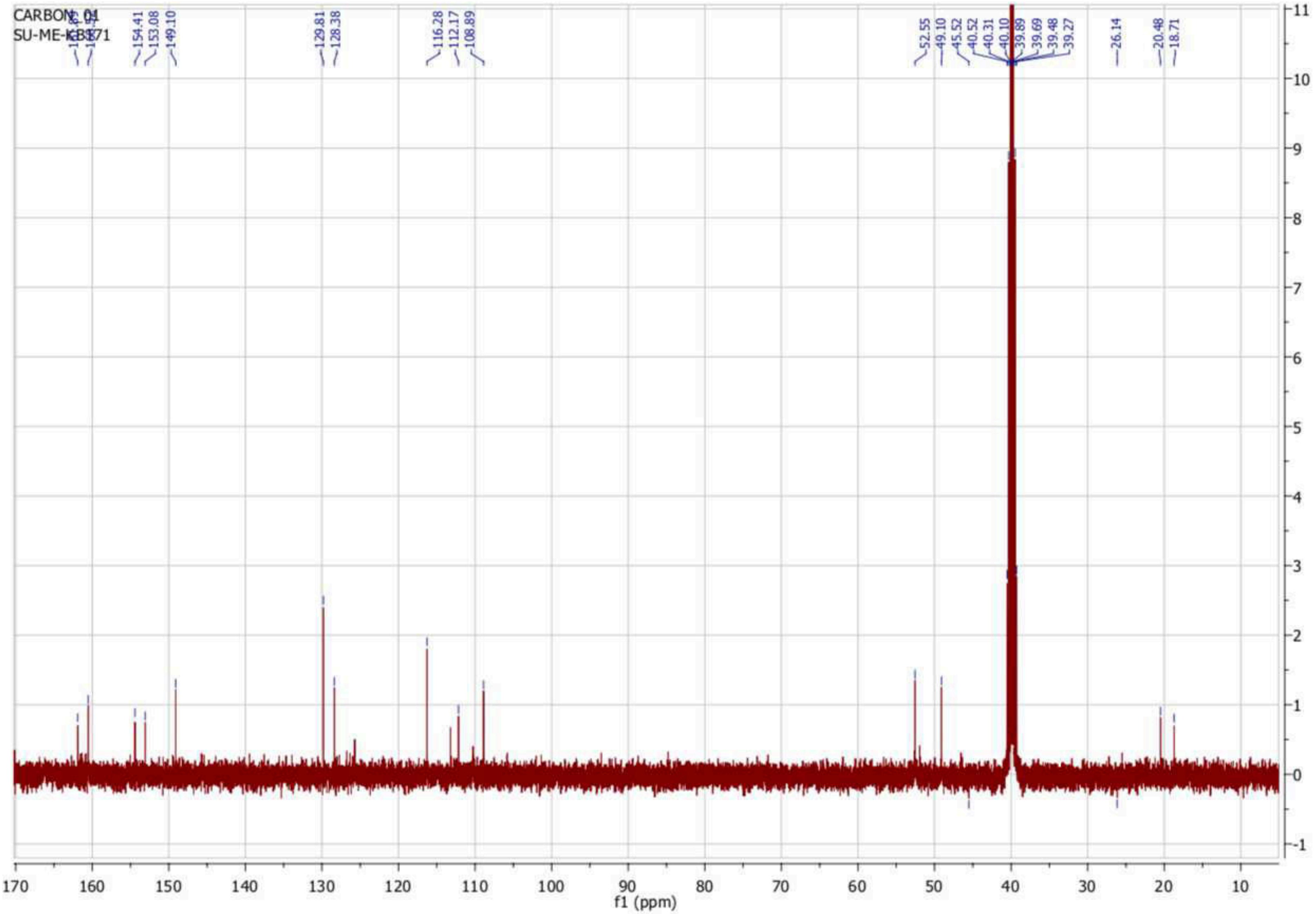

Compound 12
^{1}H NMR

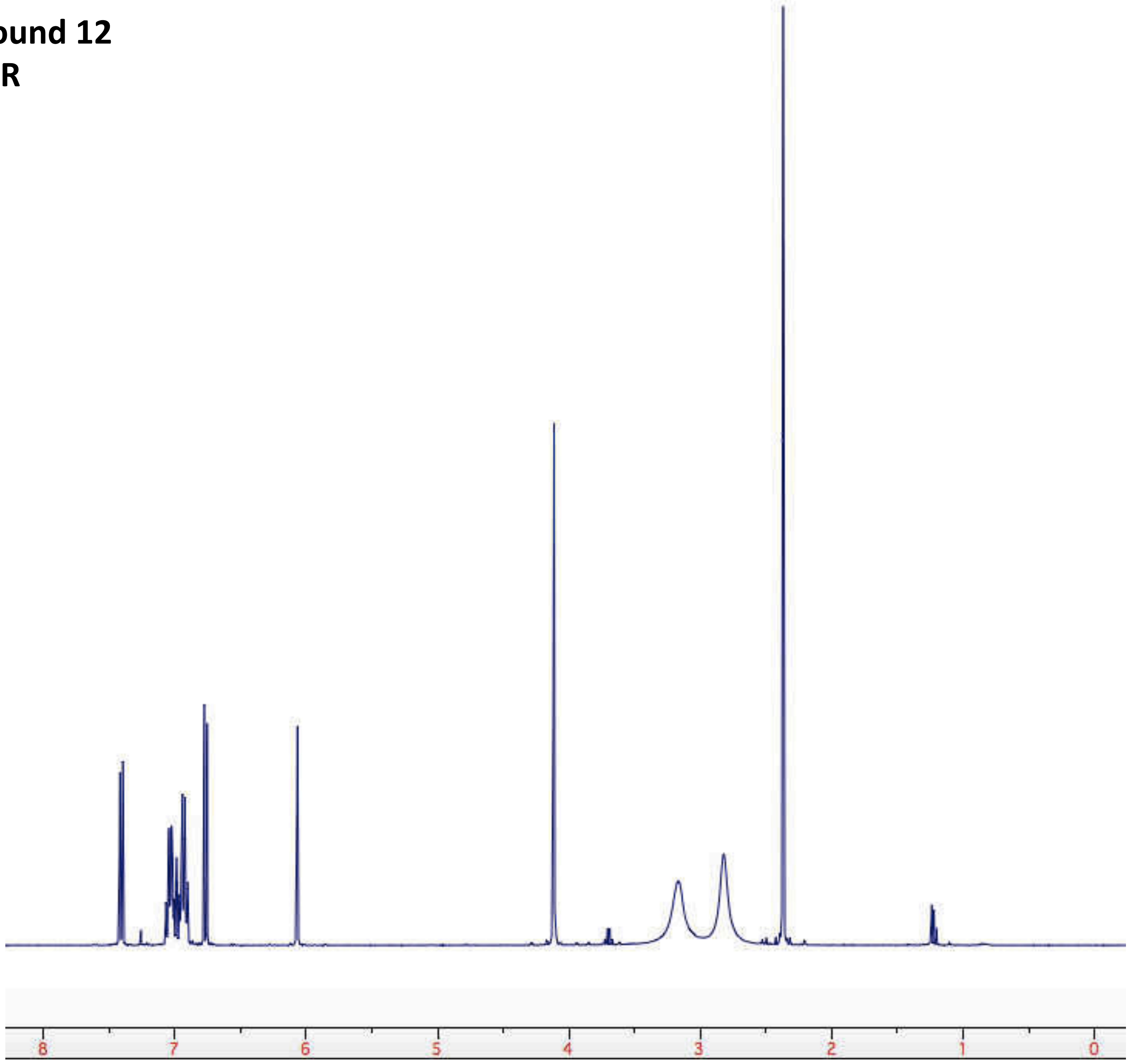

Compound 12
^{13}C NMR

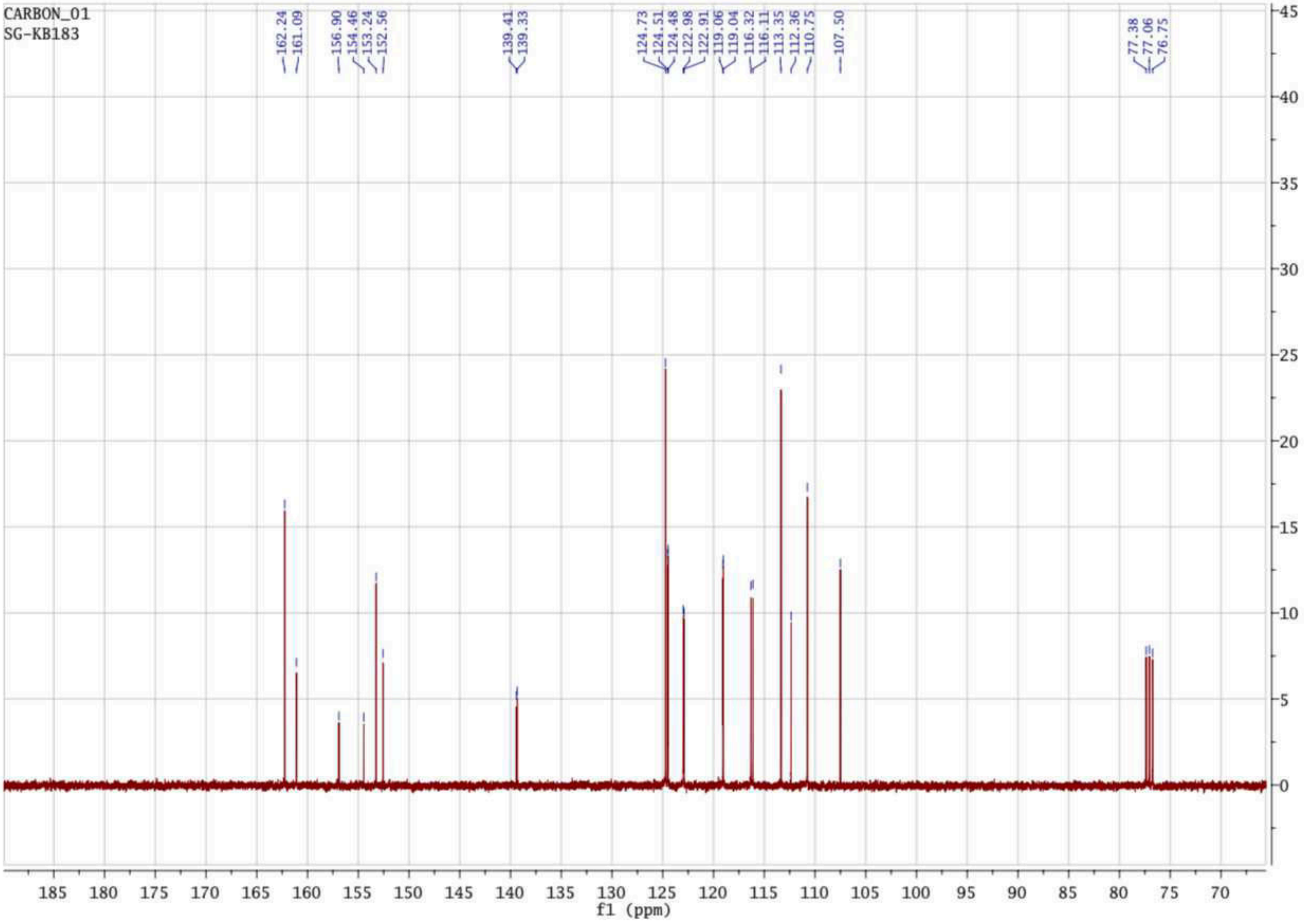

Compound 13
^{1}H NMR

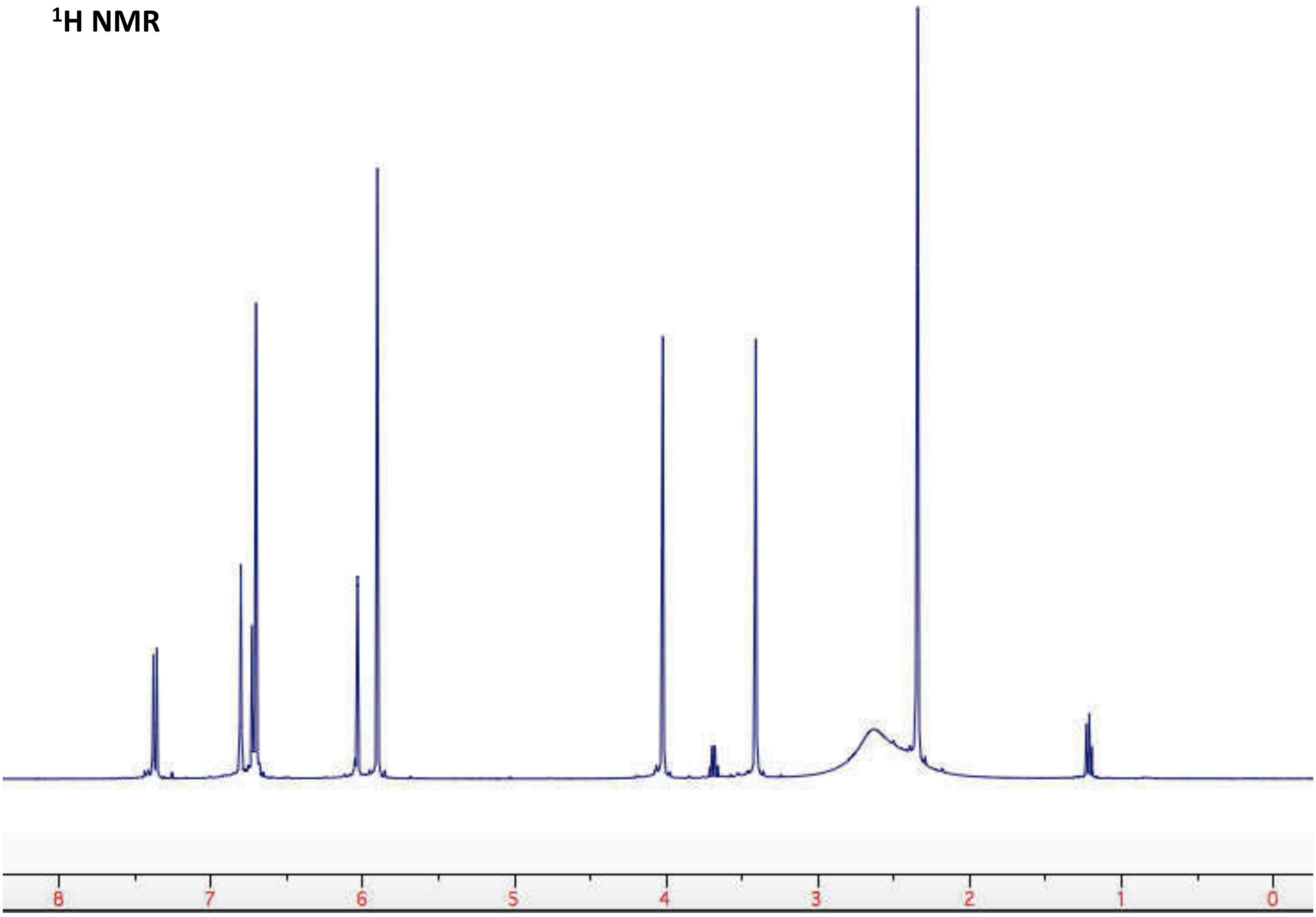

Compound 13
^{13}C NMR

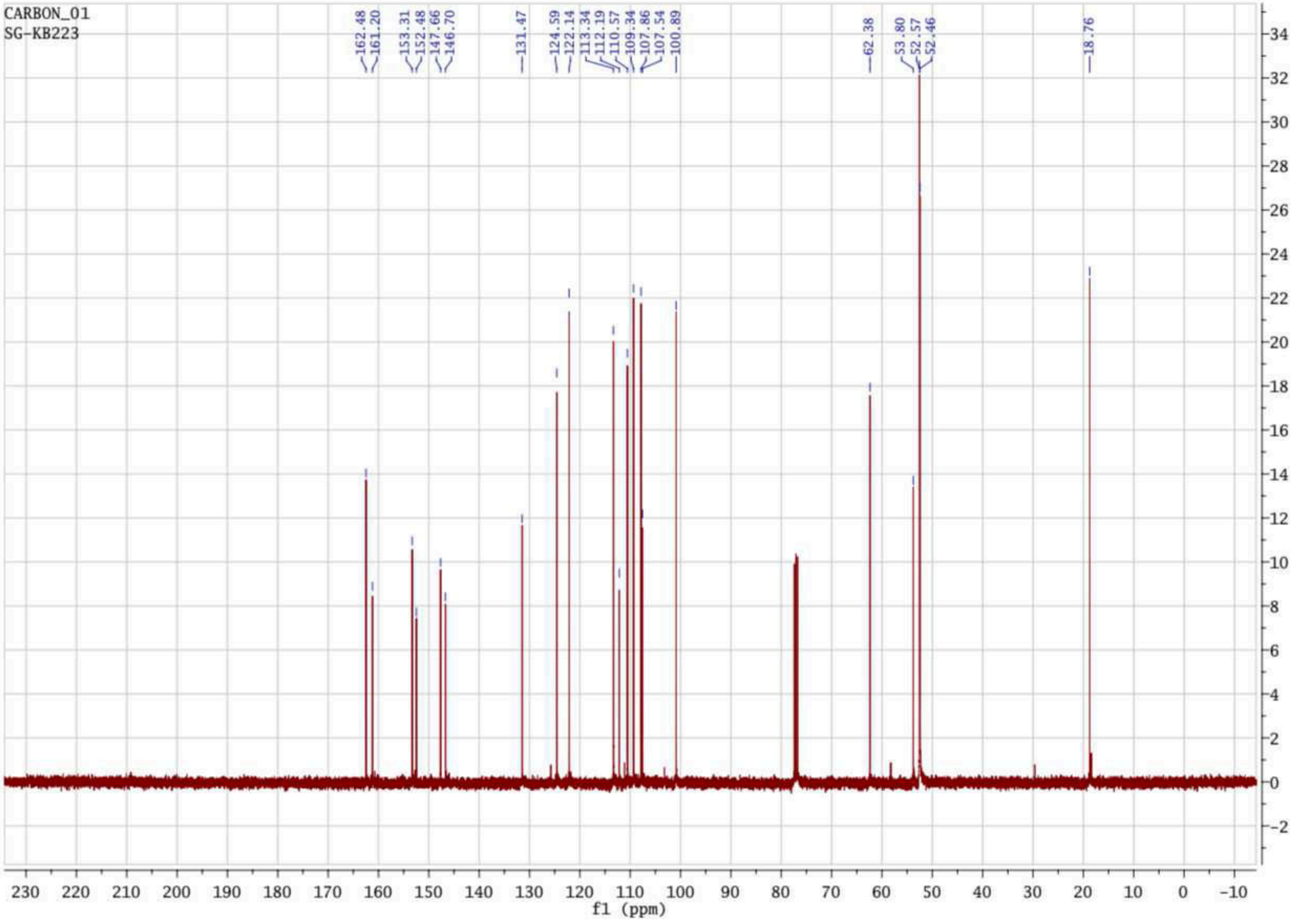

Supplement: Supplementary file 1 [file ijms-20-01208-s001.pdf]
